# Association between antidepressant use and delirium in older adults: an analysis of the World Health Organization’s global pharmacovigilance database

**DOI:** 10.1186/s12877-024-05022-0

**Published:** 2024-07-12

**Authors:** Elise-Marie Minoc, Cédric Villain, Soumia Benbrika, Basile Chrétien, Pablo Descatoire, Marie Heraudeau, Marion Sassier, Mélissa Pierre, Olivier Martinaud, Charles Dolladille, Véronique Lelong-Boulouard

**Affiliations:** 1 Geriatric Department, CHU de Caen, Avenue de La Côte de Nacre, 14000 Caen, France; 2 Normandie Univ, UNICAEN, INSERM COMETE, U1075, F-14000 Caen, France; 3 Pharmacology Department, CHU de Caen, 14000 Caen, France; 4 Normandie Univ, UNICAEN, INSERM, ANTICIPE, U1086, F-14000 Caen, France; 5 Psychiatric Department, CHU de Caen, 14000 Caen, France; 6 Neurology Department, CHU de Caen, 14000 Caen, France; 7 Normandie Univ, UNICAEN, INSERM, UMR 1077, 14000 Caen, France

**Keywords:** Antidepressants, Delirium, Older adults

## Abstract

**Background:**

Psychoactive drugs frequently cause delirium adverse events in older adults. However, few data on the relationship between antidepressants and delirium are available. Here, we investigated the association between antidepressant prescription and pharmacovigilance reports of delirium in older adults.

**Methods:**

Using the World Health Organization’s VigiBase® global pharmacovigilance database from 1967 to 2022, we performed a disproportionality analysis in order to probe the putative associations between each antidepressant class (non-selective monoamine reuptake inhibitors (NSMRIs), selective serotonin reuptake inhibitors (SSRIs), serotonin-norepinephrine reuptake inhibitors (SNRIs), monoamine oxidase inhibitors (MAOIs), alpha-2-adrenergic receptor antagonists, and other antidepressants) and reports of delirium in people aged 65 or over. We calculated the reporting odds ratios (r-OR) and their 95% confidence interval ([95%CI]) with logistic regression models before and after adjustment for confounding factors. Secondary analyses were performed for each drug and within each class by age group (65-74, and 75 and over). We also studied the reports of concomitant delirium and hyponatremia.

**Results:**

Our main analysis included 87,524 cases of delirium. After adjustment for confounders, a significant association was found between delirium and all antidepressant classes other than SNRIs. Intraclass disparities were found for the association between the most frequently prescribed antidepressants and reports of delirium. An elevated risk of reports of concomitant delirium and hyponatremia was found for SSRIs (4.46 [4.01-4.96]), SNRIs (1.25 [1.07-1.46]), MAOIs (1.72 [1.41-2.09]), and the “other antidepressants” class (1.47 [1.30-1.65]).

**Conclusions:**

There was a significant association between reports of delirium and antidepressant classes (other than SNRIs). However, this association varied from one drug to another within a given antidepressant class. Moreover, this association could not always be explained by antidepressant-induced hyponatremia.

**Supplementary Information:**

The online version contains supplementary material available at 10.1186/s12877-024-05022-0.

## Background

The reported incidence of delirium in hospitalized older adults ranges from 25 to 56% [1], and the occurrence of delirium is associated with elevated morbidity and mortality rates and higher health costs [2]. However, it has been shown that 30-40% of cases of delirium could be prevented [2]. Thus, the establishment of preventive measures is a key public health objective that could help to improve the mental health of older adults and reduce the costs associated with managing delirium.

Adverse drug events (ADEs) are major risk factors for the occurrence of delirium. It is estimated that 12-39% of cases of delirium are attributable to an ADE in general and to psychoactive drugs in particular [3]. In contrast to the well-documented association between delirium and the use of antipsychotics and benzodiazepines [3], the association with antidepressant use has been seldom studied. In fact, some antidepressants (such as non-selective monoamine reuptake inhibitors (NSMRIs)) have anticholinergic properties [4] and others (such as selective serotonin reuptake inhibitors (SSRIs) and serotonin norepinephrine reuptake inhibitors (SNRIs)) can trigger hyponatremia [5]; both of these properties are risk factors for delirium [4, 5]. Due to multimorbidity, polypharmacy (including drug interactions), and aging-related changes in drug pharmacokinetics and pharmacodynamics, antidepressants might accentuate the risk of delirium in older patients [6].

Depression is known to predispose to delirium [1] and so is a public health issue [7]. Furthermore, depression is common in patients with cognitive impairment, which itself is a major risk factor for delirium [1]. Untreated depression, the wrong choice of antidepressants, or poorly conducted treatment might impede the resolution of concomitant somatic problems, increase the time spent in hospital, augment the risk of suicide, and/or worsen treatment compliance among older patients [7]. Therefore, the treatment of depression in older adults is often challenging; the goal is to effectively relieve the depression while avoiding ADEs like delirium.

To the best of our knowledge, there are no literature data on the risk of delirium associated with the use of various antidepressant classes in older adults. We hypothesized that delirium is more strongly associated with antidepressants with anticholinergic properties (e.g. NSMRIs) or hyponatremic properties (e.g. SSRIs and SNRIs) than with other antidepressant classes. We then studied these putative associations by analyzing data from the World Health Organization (WHO)’s VigiBase® global pharmacovigilance database.

## Methods

### Study design, setting, and population

We conducted an international, retrospective, pharmacovigilance disproportionality analysis using the WHO’s VigiBase® pharmacovigilance database. VigiBase® contains more than 30 million individual case safety reports received from 160 members countries since 1967.

We used included VigiBase® data from the database’s inception to March 1st, 2022. We restricted our analysis to the individual case safety reports on people aged 65 or over. The study protocol was registered at ClinicalTrials.gov (NCT05356078). In line with the French legislation on retrospective, anonymized studies of routine medical practice (MR-004), and in accordance with the European regulation of April 27, 2016 on the protection of individuals with regard to the processing of data to personal character, the study protocol was approved by a hospital committee (C.L.E.R.S Comité Local d’Ethique de la Recherche en Santé) with competency for research not requiring authorization by an Institutional Review Board (University of Caen Normandy (Caen, France); reference: 2646, dated July 15th, 2021).

### Variables

Each individual case safety report include administrative data (country, type of report, and type of reporter), sociodemographic data (age and sex), the time to the onset of the ADE, the outcome (coded according to the Medical Dictionary for Regulatory Activities (MedDRA) version 24.0), the WHO causality assessment, and the drug(s) involved (drug name, start and stop dates, time to onset, indication, dose, dechallenge, and rechallenge).

In VigiBase®, drugs are coded using the WHODrug Global dictionary. Antidepressant classes were based on the Anatomical Therapeutic Chemical (ATC) hierarchical classification and classified into NSMRIs, SSRIs, SNRIs, monoamine oxidase inhibitors (MAOIs), alpha-2-adrenergic receptor antagonists, and other antidepressants (for details, see Supplementary Table 1). Most studies included SNRIs and alpha-2-adrenergic receptor antagonists in an "other antidepressants" class; however, in view of their specific pharmacodynamic properties and their frequency of use, we decided to consider these two classes in their own right [5, 8]. We also noted the most frequently prescribed antidepressant drugs within each class, defined as those mentioned in more than 1000 reports (regardless of the type of ADE) in VigiBase®.

ADEs were coded according to the MedDRA terminology. In the present study, the event “delirium” encompassed the MedDRA terms “Delirium”, “Confusional state” and “Disorientation”. A concomitant delirium-hyponatremia event was defined as a report in which delirium (as defined above) and hyponatremia at the same time were reported.

We selected all types of reports, regardless of whether the antidepressant was suspected to be responsible for delirium, concomitantly prescribed with another suspected drug, or thought to be interacting with another drug.

### Outcomes

The primary outcome was the association between antidepressant use and reports of delirium among people aged 65 or over. The secondary outcomes included (i) the association between the most frequently prescribed antidepressants and reports of delirium, (ii) the association between antidepressant classes and concomitant delirium-hyponatremia events, and (iii) the same associations by age class (65 to 74 vs. 75 and over).

### Statistical methods

A case/non-case disproportionality analysis was used to probe the effect of antidepressant prescription on reports of delirium; this type of analysis has been described in detail previously [9]. The case/non-case disproportionality method is recommended for detecting a signal for an association between a drug and an adverse event in a pharmacovigilance database. A signal is present when the number of reports of an adverse event is greater than expected. This is referred to as a disproportionate reporting rate of an adverse event, relative to others. In the present study, a signal corresponded to a statistically significant difference in the distribution of cases of delirium related to antidepressants or classes of antidepressant vs. cases of delirium related to drugs other than antidepressants. The strength of the disproportionality was quantified as the reporting odds ratio (r-OR) and their 95% confidence interval ([95%CI]) estimated with univariate and multivariate logistic regression models. An r-OR was considered statistically significant if it was higher than 1 and if the lower boundary of its 95%CI did not include 1. An r-OR lower than 1 was considered statistically unsignificant, as the method was unable to detect the absence of a signal.

Disproportionality analyses typically include positive controls (i.e. drugs or drugs classes established to trigger the ADE of interest) and negative controls (i.e. drugs that are not known to trigger the ADE of interest); if the obtained results and the expected results are consistent, major sources of bias are likely to be absent. In the present multivariate analysis, we chose natural opium alkaloids (ATC N02AA) as positive controls and bisphosphonates (ATC M05BA) as negative controls [3].

Our models were adjusted for potential confounders: age class (65-74, 75 and over), sex, geographic region, and the major potentially associated prescriptions of drugs and illnesses reported in the literature as inducing delirium (opioids (ATC N02), antipsychotics (ATC N05A), anxiolytics (ATC N05B), and hypnotics (ATC N05C), constipation, acute urinary retention, alcohol use, unspecified infections, drug misuse, dementia, dehydration, hyponatremia (“hyponatraemia”, according to the spelling used in MedDRA), anticholinergic syndrome, hypoglycemia (“hypoglycaemia”, according to the spelling used in MedDRA), seizure disorder, drug abuse, drug dependence, drug withdrawal, central nervous system vascular disorders, hearing impairment, and visual impairment [2, 3, 10–12]; for details, see Supplementary Table 2. We studied the collinearity of our final models by computing the variance-inflation factors.

Sensitivity analyses were conducted on a multivariate model for the primary outcome by using three different definitions of delirium: (i) “Delirium” or “Confusional state”; (ii) “Delirium” or “Confusional state” or “Disorientation” or “Circadian rhythm sleep disorder”; and (iii) “Delirium” or “Confusional state” or “Disorientation” or “Circadian rhythm sleep disorder” or “Hallucinations”.

In analyses of pharmacovigilance databases, it is sometimes not possible to assess certain variables because the latter data are missing or because the patient did not meet certain clinical or pharmacologic criteria. In the present study, the person’s age was specified for all reports; hence, sex was the only variable for which missing data could have been imputed. As less than 1% of the data for this variable were missing, we decided not to impute them and so performed a complete case analysis.

All statistical analyses were performed using R software (v 4.0.2, R Studio v1.4.1717) and its packages rlang, dplyr, stats, base, fst, data.table, magrittr, openxlsx, carData, car, grid, and checkmate [13].

## Results

### Descriptive analysis of the study population

In VigiBase®, we found 87,524 reports of delirium in people aged 65 and over (Table 1). The 65-74 and 75 + age groups accounted for 41% and 59% of the reports, respectively. 54% of the reports concerned women. Of the people presenting delirium, 22% were being treated with antidepressants; SSRIs: 7%; other antidepressants: 7%; NSMRIs: 3%; SNRIs: 3%; alpha-2-adrenergic receptor antagonists: 2%; MAOIs: < 1%. It is also noteworthy that 34% of patients with delirium were being treated with opioids, and 7% suffered from an unspecified infection.

**Table 1:**
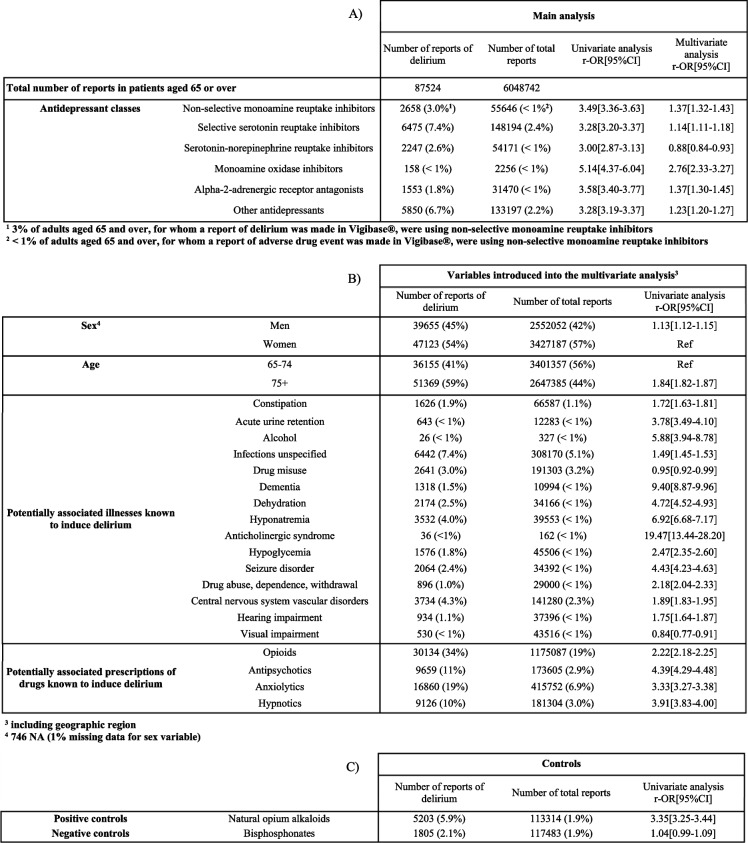
The risk of reports of delirium in older adults, and characteristics of the study population

### Primary outcome

**Fig. 1:**
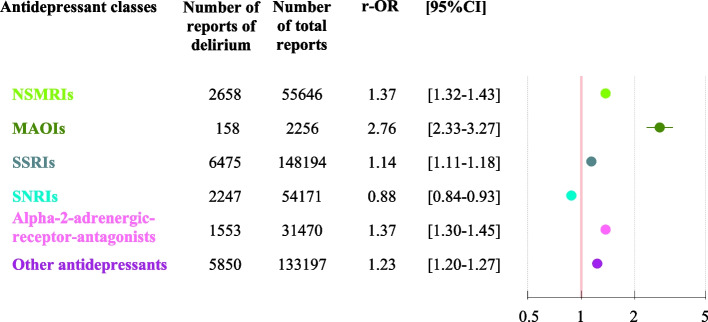
Multivariate analysis of the association between antidepressant classes and reports of delirium in older adults

The associations between antidepressant classes and delirium are shown in Table 1 and Fig. 1. No collinearity was found in our final models (Supplementary Table 3). There was a significant association between each antidepressant class and delirium, with the exception of the SNRIs (Fig. 1).

### Secondary outcomes

#### Associations between the most frequently prescribed antidepressants and reports of delirium in patients aged 65 or over

**Fig. 2:**
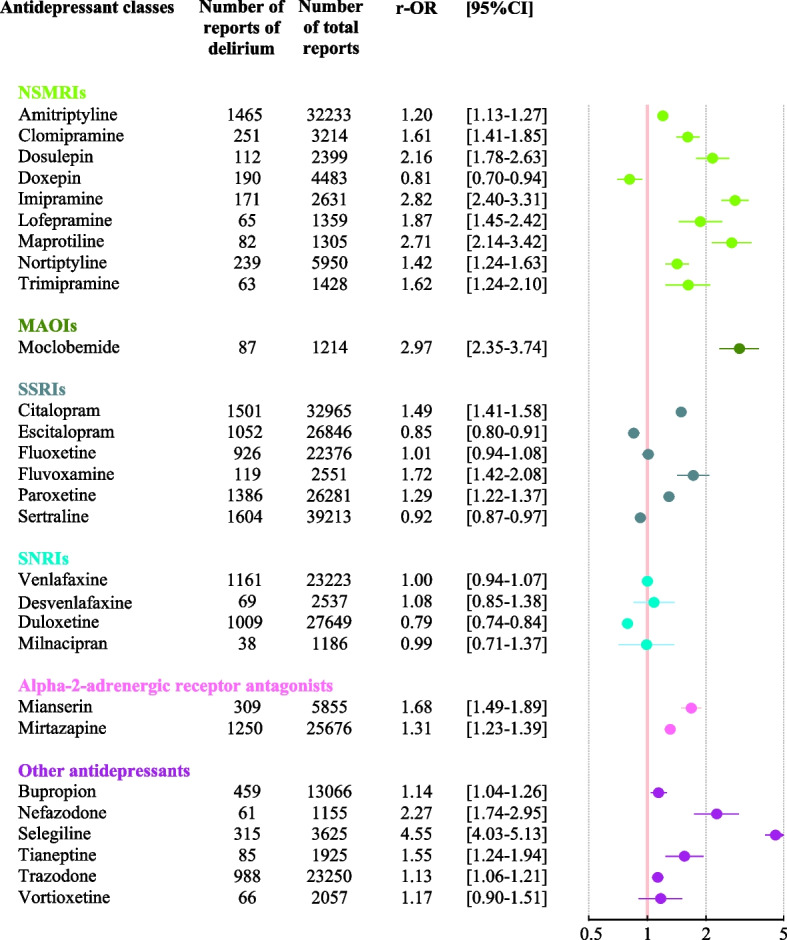
Multivariate analysis of the association between the most frequently prescribed antidepressants and reports of delirium in older adults

We observed a significant, positive association with reports of delirium for all NSMRIs other than doxepin (Fig. 2). Moclobemide was the only MAOI to be studied; we observed a significant association with reports of delirium (2.97 [2.35-3.74]). When considering the SSRIs, reports of delirium were significantly associated with citalopram (1.49 [1.41-1.58]), fluvoxamine (1.72 [1.42-2.08]), and paroxetine (1.29 [1.22-1.37]) but not fluoxetine, escitalopram, or sertraline. Within the SNRI class, no associations with reports of delirium were found. Within the alpha-2-adrenergic receptor antagonist class, mianserin (1.68 [1.49-1.89]) and mirtazapine (1.31 [1.23-1.39]) were associated with reports of delirium. Within the “other antidepressants” class, a significant association with reports of delirium was found for all the studied drugs other than vortioxetine.

#### Associations between antidepressant classes and concomitant delirium and hyponatremia

**Fig. 3:**
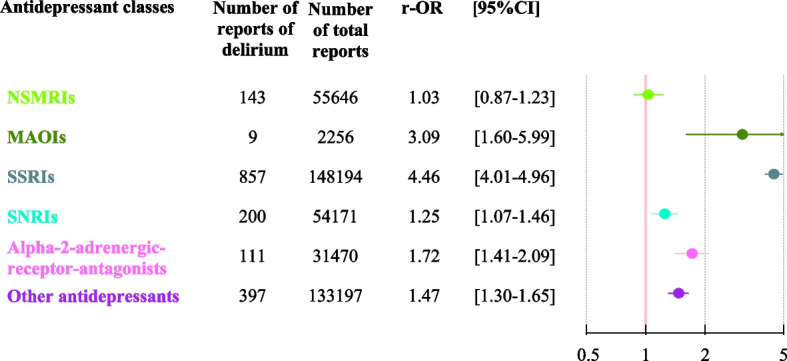
Multivariate analysis of the association between antidepressant classes and reports of concomitant delirium and hyponatremia in older adults

All the antidepressant classes other than NSMRIs were significantly associated with reports of concomitant delirium and hyponatremia (Fig. 3 and Supplementary Table 4). The strongest association was found for SSRIs (4.46 [4.01-4.96]).

#### Associations by age group

**Fig. 4:**
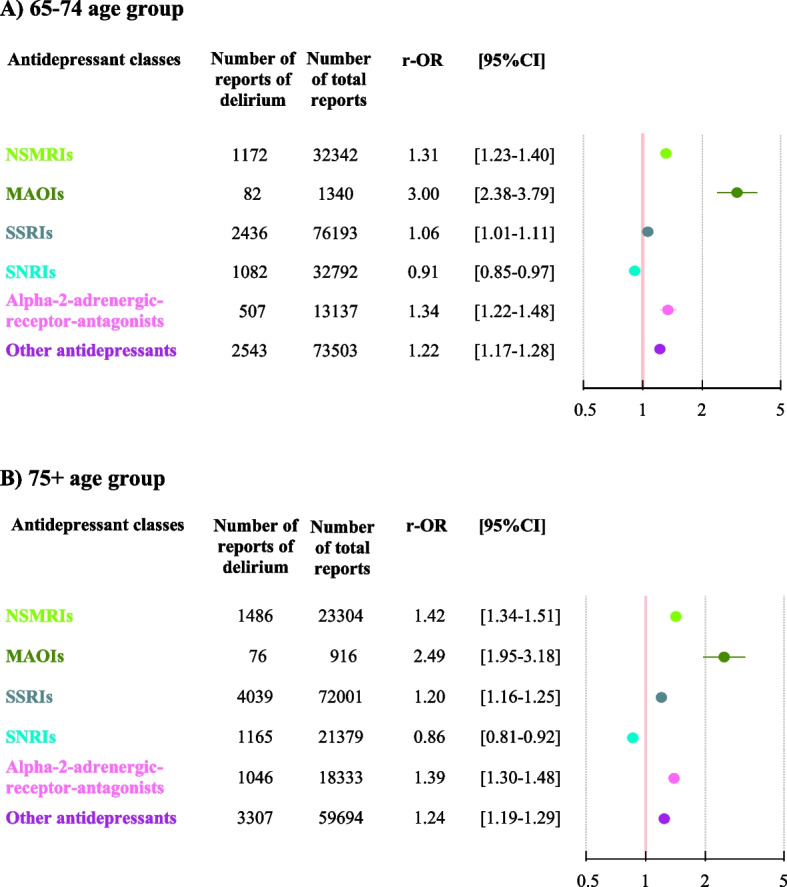
Multivariate analysis of the association between antidepressant classes and reports of delirium, by age group

The associations between antidepressant classes and delirium reporting for the 65-74 and 75 + age groups were similar to those of the main analysis (Fig. 4 and Supplementary Tables 5 and 6).

### Sensitivity analyses

The results of sensitivity analyses using the various definitions of delirium were consistent with the those of the primary analysis (Supplementary Table 7).

### Positive and negative controls

As expected, the positive control was significantly and positively association with reports of delirium (3.35 [3.25-3.44]) and the negative control was not (1.04 [0.99-1.09]) (Table 1).

## Discussion

The overall objective of the present study was to investigate the association between antidepressant classes and reports of delirium in VigiBase®. To the best of our knowledge, the present study population of older adults, for whom an adverse drug event had been reported, is the largest yet studied in this respect. After adjusting for a large number of potential confounders, we found that all antidepressant classes other than SNRIs were associated with an elevated risk of delirium reporting in older patients. These results by age class did not differ markedly from the overall results. We also observed that within a given antidepressant class, the risk varied from one drug to another. All antidepressant classes other than NSMRIs were associated with reports of concomitant delirium and hyponatremia.

NSMRIs are known to have anticholinergic and sedative properties via their affinities for muscarinic acetylcholine receptors and histamine H1 receptors, respectively [14]. Both of these properties are known to be precipitating risk factors for delirium [4, 15]. Here, we indeed observed a significant, positive association between NSMRIs and reports of delirium. This association was found for all NSMRIs other than doxepin, which has a similar mechanism of action but a lower affinity for muscarinic receptors (and thus a weaker anticholinergic effect) than other drugs in this class [16]. Due to its high affinity for a subtype of histamine H1 receptor, doxepin is also used to manage chronic urticaria [17]. Thus, the characteristics of patients treated with doxepin might differ from those of other patients treated with NSMRIs – particularly in terms of psychiatric comorbidities like depression, which is associated with a greater likelihood of reports of delirium.

We also found a significant association between the use of alpha-2-adrenergic receptor antagonists and reports of delirium. Although alpha-2-adrenergic receptor antagonists do not have any anticholinergic activity [14], antagonism of histamine H1 receptors should also be considered for this class of antidepressant. mianserin and mirtazapine have a high affinity for histamine H1 receptors (as do first-generation antihistamines) [18, 19]. Thus, the sedative properties of histamine H1 receptor antagonists might also explain the elevated risk of reports of delirium associated with alpha-2-adrenergic receptor antagonists [15].

Most SSRIs lack anticholinergic or antihistaminic properties [14], although paroxetine and fluvoxamine both have anticholinergic activity [4]. It is noteworthy that in the present study, both paroxetine and fluvoxamine were associated with the reports of delirium. This finding strengthens the hypothesis whereby the elevated risk of delirium reporting associated with antidepressants is partly explained by anticholinergic mechanisms. Moreover, the results for escitalopram vs. citalopram were discordant. It should be borne in mind that citalopram is a 1:1 racemate of R- and S-enantiomers, whereas escitalopram is S-citalopram [20]. It has been shown that R-citalopram limits the therapeutic efficacy and delays the onset of action of S-citalopram; this explains why the required doses are higher for citalopram than for escitalopram [20]. It has also been shown in animals that the presence of the R-enantiomer in citalopram can produce a paradoxical anxiogenic effect above a certain concentration threshold [20]. The R- and S-enantiomers therefore differ in their pharmacokinetic, pharmacodynamic and clinical properties. The difference between citalopram and escitalopram observed in our study might also be due to the preferential use of citalopram in severe forms of depression, for which parenteral administration is perhaps superior to oral administration [21]. One can hypothesize that the people taking citalopram had more severe depression, refused oral administration, or had other behavioral disturbances; this would constitute a confounding bias and might explain the significant association between reports of delirium and citalopram [22] but not escitalopram.

Interestingly, SNRIs were the only antidepressant class not associated with reports of delirium in our study. Although the r-OR for SNRIs was < 1 with its higher 95%CI boundary < 1, it cannot be interpreted as a protective signal, in the setting of disproportionality analysis. This lack of an association might be due to the drugs’ pharmacodynamic properties. Indeed, SNRIs have a more selective action on the reuptake of serotonin and noradrenaline and are not linked to adverse effects caused by cholinergic and histamine receptor blockades [23]. One can hypothesize that these properties of the SNRIs can explain (at least in part) the lack of an association with the occurrence of delirium in older people with high inter-individual pharmacodynamic variability [6].

Here, we found that concomitant delirium and hyponatremia were associated with the use of SSRIs, SNRIs, alpha-2-adrenergic receptor antagonists, and MAOIs. These findings are consistent with the literature data on an association between SSRIs/SNRIs and hyponatremia [5]. The literature data on alpha-2-adrenergic receptor antagonists are somewhat contradictory but tend to show an association with hyponatremia [24]. Significant associations with SSRIs, SNRIs and alpha-2-adrenergic receptor antagonists might be due to the drugs’ action on certain serotonin receptors, which would lead to inappropriate antidiuretic hormone secretion and then hyponatremia [24]. Our results for MAOIs are consistent with an analysis of the French national pharmacovigilance database [25]. However, the number of reported events was small in both studies (only 9 here, for example); this lack of statistical power and the conflicting literature results make it hard to draw firm conclusions in this respect [5]. One can nevertheless suspect that the occurrence of delirium is mediated partly by the induction of hyponatremia by antidepressant drugs from these four classes.

In contrast, we did not find a significant association between NSMRIs and concomitant delirium and hyponatremia in our study. Nevertheless, an association between some NSMRIs and the occurrence of hyponatremia has been reported [26]. Even though NSMRIs are less likely to cause hyponatremia than SSRIs [5], they can still induce inappropriate antidiuretic hormone secretion [26]. Hence, hyponatremia is unlikely to be the main mechanism in NSMRI-induced delirium. Likewise, it would have been interesting to study the association between antidepressant classes and anticholinergic syndrome. However, the very small number of cases reported in VigiBase® prevented us from performing a robust analysis.

The “other antidepressants” class was also associated with reports of delirium and reports of concomitant delirium and hyponatremia. The antidepressants classified in this class have very different pharmacodynamic properties [8] and so the results for each drug should be interpreted with caution, due to a possible lack of statistical power. There are only a few literature data (from case reports and a study of the French national pharmacovigilance database) in support of our present findings [25, 27].

The present study had a number of strengths, most of which were due to its statistical methodology. Firstly, VigiBase® includes 30 million reports on ADEs from more than 160 countries and thus provides a large sample of older adults with reported delirium. Secondly, disproportionality analysis is a well-established method for detecting signals in pharmacovigilance safety research and post-marketing surveillance [28]. Lastly, our multivariate analysis and our use of positive and negative controls reduced potential sources of bias and enhanced the validity of our hypothesis [9].

### Study limitations

Although pharmacovigilance analysis can detect safety signals and generate hypotheses, it cannot alone provide evidence of causal associations; this constituted a first limitation [28]. Moreover, the diagnosis of delirium can be challenging, due to a variety of presentations and the probable under-reporting of hypoactive forms [12]. However, in order to limit possible classification bias in our study, we defined cases of delirium by reference to the MedDRA terms “Delirium”, “Confusional state” and “Disorientation”. Indeed, our chosen definition was shown to be robust in comparative sensitivity analyses.

Secondly, we were not able to check the diagnoses reported in VigiBase®. Thirdly, the individual case safety reports did not all have details of the age, drug dose, time of onset, comorbid conditions, and concomitant medications [28]. For example, the prevalence of dementia in Vigibase® for adults aged 65 and over was 0.2% (10994 reported cases out of 6048742 adults aged 65 and over) but (according to the WHO) is about 5-8% in people aged 60 and over [29]. Lastly, our focus on delirium prevented us from drawing conclusion about the overall iatrogenic burden of antidepressant drugs or each antidepressant class.

## Conclusions

We observed a significant association between most antidepressant classes and reports of delirium in VigiBase®. This likelihood of reports was greater for antidepressants with high affinity for histamine H1 receptors and/or muscarinic receptors than for antidepressants lacking these properties. Our results might suggest that the use of SNRIs (venlafaxine, desvenlafaxine, duloxetine, milnacipran) and certain SSRIs (escitalopram, sertraline, fluoxetine) as well as doxepin and vortioxetine might be safer in older people at a high risk of delirium, such as those with cognitive impairment. However, further preclinical and clinical studies (e.g. animal models, and functional imaging in humans) of the antidepressants’ mechanisms of action in older people will be necessary to consider practical applications of our results.

### Supplementary Information

Supplementary Material 1.

## Data Availability

The study data are the property of the World Health Organization, the corresponding author has access to the data extraction and the R script used to analyse it.

## Abbreviations

NSMRIs: Non-selective monoamine reuptake inhibitors
SSRIs: Selective serotonin reuptake inhibitors
SNRIs: Serotonin-norepinephrine reuptake inhibitors
MAOIs: Monoamine oxidase inhibitors
r-OR: Reporting odds ratios
[95%CI]: 95% Confidence interval
ADEs: Adverse drug events
WHO: World Health Organization
MedDRA: Medical Dictionary for Regulatory Activities
ATC: Anatomical Therapeutic Chemical
