# Supplementary material for: Association between antidepressant use and delirium in older adults: an analysis of the World Health Organization’s global pharmacovigilance database
Source: BMC Geriatr. 2024 Jul 12;24:600. doi: 10.1186/s12877-024-05022-0 (PMC11241964; doi:10.1186/s12877-024-05022-0)
Supplement: Supplementary file 1 — Supplementary Material 1. [file 12877_2024_5022_MOESM1_ESM.pdf]

Supplementary Table 1 – List of antidepressants, by class

Non-selective monoamine reuptake inhibitors (NSMRIs)	amineptine, amitriptyline, amoxapine, butriptyline, clocapramine, clomacran, clomipramine, demexiptiline, desipramine, desmethylclomipramine, dibenzepin, diclofensine, dimetacrine, dosulepin, doxepin, imipramine, imipraminoxide, iprindole, liafensine, lofepramine, maprotiline, melitracen, metapramine, nortriptyline, noxiptiline, opipramol, oxaprotiline, pipofezine, protriptyline, quinupramine, trimipramine
Monoamine oxidase inhibitors (MAOIs)	amiflamine, befloxatone, brofaromine, moclobemide, pirlindole, toloxatone, iproclozide, iproniazid, isocarboxazid, mebanazine, nialamide, phenelzine, pheniprazine, safrazine, tranylcypromine
Selective serotonin reuptake inhibitors (SSRIs)	alaproclate, cianopramine, citalopram, escitalopram, etoperidone, femoxetine, fluoxetine, fluvoxamine, indalpine, paroxetine, seproxetine, sertraline, zimeldine
Serotonin-norepinephrine reuptake inhibitors (SNRIs)	venlafaxine, desvenlafaxine, duloxetine, milnacipran, levomilnacipran
Alpha-2-adrenergic receptor antagonists	mianserin, mirtazapine, setiptiline
Other antidepressants	ademetionine, agomelatine, amesergide, amibegron, amitifadine, ansofaxine, apimostinel, basimglurant, beloxepin, bifemelane, brexanolone, bupropion, cutamesine, decoglurant, dexmecamylamine, edivoxetine, eptapirone, esketamine, esreboxetine, gepirone, indeloxazine, medifoxamine, mefexamide, minaprine, nefazodone, nitroxazepine, nomifensine, oxaflozane, oxitriptan, pivagabine, prazitone, pyridoxine, rapastinel, reboxetine, rislenemdaz, rubidium, sceletium, selegiline, tedatioxetine, tianeptine, trazodone, tryptophan, verucerfont, vilazodone, viloxazine, vortioxetine

Supplementary Table 2 – Preferred terms for illnesses introduced into the multivariate model

The major potentially associated illnesses known to induce delirium and that were introduced into the multivariate model	Corresponding preferred terms (PT) from the MedDRA classification
Constipation	Constipation (PT), Faecaloma (PT)
Acute urinary retention	Urinary retention (PT)
Alcohol use	Alcohol abuse (PT), Alcohol poisoning (PT), Alcohol problem (PT), Alcohol use disorder (PT), Alcoholism (PT)
Unspecified infections	Abdominal abscess (PT), Abdominal hernia infection (PT), Abdominal infection (PT), Abdominal wall abscess (PT), Abdominal wall infection (PT), Abscess intestinal (PT), Anal abscess (PT), Anal fistula infection (PT), Anal infection (PT), Anal papillitis (PT), Anorectal infection (PT), Appendiceal abscess (PT), Appendicitis (PT), Appendicitis perforated (PT), Biloma infected (PT), Colonic abscess (PT), Colostomy infection (PT), Complicated appendicitis (PT), Diarrhoea infectious (PT), Diarrhoea infectious neonatal (PT), Diverticulitis (PT), Diverticulitis intestinal haemorrhagic (PT), Diverticulitis intestinal perforated (PT), Douglas' abscess (PT), Dysentery (PT), Enteritis infectious (PT), Enterocolitis infectious (PT), Focal peritonitis (PT), Gastric infection (PT), Gastroenteritis (PT), Gastrointestinal infection (PT), Haemorrhoid infection (PT), Infected large intestinal ulcer (PT), Infective mesenteric panniculitis (PT), Intestinal fistula infection (PT), Large intestine infection (PT), Mesenteric abscess (PT), Oesophageal abscess (PT), Oesophageal infection (PT), Pancreas infection (PT), Pancreatic abscess (PT), Parasitic gastroenteritis (PT), Perihepatic abscess (PT), Perirectal abscess (PT), Peritoneal abscess (PT), Peritonitis (PT), Pyloric abscess (PT), Rectal abscess (PT), Retroperitoneal abscess (PT), Retroperitoneal infection (PT), Retroperitonitis (PT), Stump appendicitis (PT), Subdiaphragmatic abscess (PT), Abscess jaw (PT), Administration site joint infection (PT), Application site joint infection (PT), Arthritis infective (PT), Bone abscess (PT), Bursitis infective (PT), Fracture infection (PT), Infected bunion (PT), Infected gouty tophus (PT), Infective chondritis (PT), Infective periostitis (PT), Infective spondylitis (PT), Infusion site joint infection (PT), Injection site joint infection (PT), Intervertebral discitis (PT), Joint abscess (PT), Medical device site joint infection (PT), Osteomyelitis (PT), Osteomyelitis acute (PT), Osteomyelitis chronic (PT), Paraspinal abscess (PT), Purulent synovitis (PT), Sternitis (PT), Subperiosteal abscess (PT), Vaccination site joint infection (PT), Breast abscess (PT), Breast discharge infected (PT), Mastitis (PT), Mastitis postpartum (PT), Nipple infection (PT), Recurrent subareolar breast abscess (PT), Acute endocarditis (PT), Cardiac infection (PT), Cardiac valve abscess (PT), Cardiac valve vegetation (PT), Endocarditis (PT), Infective pericardial effusion (PT), Myocardiac abscess (PT), Myocarditis infectious (PT), Myocarditis septic (PT), Pericarditis infective (PT), Prosthetic valve endocarditis (PT), Purulent pericarditis (PT), Septic cardiomyopathy (PT), Septic endocarditis (PT), Subacute endocarditis (PT), Brain abscess (PT), Brain empyema (PT), Cavernous sinus thrombosis (PT), Central nervous system abscess (PT), Central nervous system infection (PT), CNS ventriculitis (PT), Cranial nerve infection (PT), Dural abscess (PT), Encephalitis (PT), Encephalitis brain stem (PT), Encephalitis lethargica (PT), Encephalomyelitis (PT), Ependymitis (PT), Extradural abscess (PT), Intracranial infection (PT), Meningitis (PT), Meningitis aseptic (PT), Meningitis neonatal (PT), Myelitis (PT), Neurological infection (PT), Panencephalitis (PT), Spinal cord abscess (PT), Spinal cord infection (PT), Spinal empyema (PT), Subarachnoid abscess (PT), Subdural abscess (PT), Abscess of salivary gland (PT), Abscess oral (PT), Alveolar osteitis (PT), Dental fistula (PT), Dental gangrene (PT), Gingival abscess (PT), Gingivitis (PT), Infective glossitis (PT), Lip infection (PT), Ludwig angina (PT), Oral infection (PT), Oral pustule (PT), Papillon-Lefevre syndrome (PT), Parotid abscess (PT), Parotitis (PT), Pericoronitis (PT), Peri-implantitis (PT), Periodontal destruction (PT), Periodontitis (PT), Pulpitis dental (PT), Root canal infection (PT), Sialoadenitis (PT), Submandibular abscess (PT), Tongue abscess (PT), Tooth abscess (PT), Tooth infection (PT), Abscess of external auditory meatus (PT), Ear infection (PT), Ear lobe infection (PT), Gradenigo's syndrome (PT), Infected aural fistula (PT), Labyrinthitis (PT), Mastoid abscess (PT), Mastoid empyema (PT), Mastoiditis (PT), Myringitis (PT), Otitis externa (PT), Otitis media (PT), Otitis media acute (PT), Otitis media chronic (PT), Otosalpingitis (PT), Petrositis (PT), Abscess of eyelid (PT), Blebitis (PT), Chorioretinitis (PT), Conjunctivitis (PT), Corneal abscess (PT), Corneal infection (PT), Dacryocanaliculitis (PT), Dacryocystitis (PT), Endophthalmitis (PT), Eye abscess (PT), Eye infection (PT), Eye infection intraocular (PT), Eyelid boil (PT), Eyelid infection (PT), Hordeolum (PT), Hypopyon (PT), Infectious crystalline keratopathy (PT), Infectious iridocyclitis (PT), Infective corneal ulcer (PT), Infective episcleritis (PT), Infective iritis (PT), Infective keratitis (PT), Infective scleritis (PT), Infective uveitis (PT), Keratouveitis (PT), Lacrimal gland abscess (PT), Oculoglandular syndrome (PT), Ophthalmia neonatorum (PT), Orbital infection (PT), Periorbital abscess (PT), Periorbital infection (PT), Retinitis (PT), Vitreous abscess (PT), Vitritis infective (PT), Abortion infected (PT), Amniotic cavity infection (PT), Bartholinitis (PT), Bartholin's abscess (PT), Cervicitis (PT), Clitoris abscess (PT), Endometritis (PT), Endometritis decidual (PT), Fallopian tube abscess (PT), Funisitis (PT), Genital infection female (PT), Induced abortion infection (PT), Intrauterine infection (PT), Lochial infection (PT), Myometritis (PT), Neovaginal infection (PT), Oophoritis (PT), Ovarian abscess (PT), Parametric abscess (PT), Parametritis (PT), Pelvic inflammatory disease (PT), Post abortion infection (PT), Pregnancy related infection (PT), Puerperal infection (PT), Puerperal pyrexia (PT), Pyometra (PT), Salpingitis (PT), Salpingo-oophoritis (PT), Tubo-ovarian abscess (PT), Umbilical sepsis (PT), Uterine abscess (PT), Uterine infection (PT), Vaginal abscess (PT), Vaginal infection (PT), Vulval abscess (PT), Vulvitis (PT), Vulvovaginitis (PT), Biliary abscess (PT), Biliary sepsis (PT), Biliary tract infection (PT), Cholangitis infective (PT), Cholecystitis infective (PT), Emphysematous cholecystitis (PT), Gallbladder abscess (PT), Gallbladder empyema (PT), Hepatic cyst infection (PT), Hepatic infection (PT), Hepatitis post transfusion (PT), Hepatobiliary infection (PT), Hepatosplenic abscess (PT), Liver abscess (PT), Portal pyaemia (PT), Recurrent pyogenic cholangitis (PT), Splenic abscess (PT), Splenic infection (PT), Abscess (PT), Abscess limb (PT), Abscess rupture (PT), Abscess soft tissue (PT), Activated PI3 kinase delta syndrome (PT), Administration site abscess (PT), Administration site infection (PT), Adrenal gland abscess (PT), Adrenalitis (PT), Anastomotic infection (PT), Application site abscess (PT), Application site infection (PT), Catheter site abscess (PT), Catheter site infection (PT), Coinfection (PT), Congenital infection (PT), Cross infection (PT), Device related infection (PT), Ear, nose and throat infection (PT), Empyema (PT), Febrile infection (PT), Genital abscess (PT), Genital infection (PT), Genital ulcer syndrome (PT), Graft infection (PT), Groin abscess (PT), Groin infection (PT), Implant site abscess (PT), Implant site infection (PT), Incision site abscess (PT), Infantile septic granulomatosis (PT), Infected bite (PT), Infected cyst (PT), Infected fistula (PT), Infected metastasis (PT), Infected neoplasm (PT), Infected seroma (PT), Infection (PT), Infection in an immunocompromised host (PT), Infection masked (PT), Infection parasitic (PT), Infection reactivation (PT), Infection susceptibility increased (PT), Infectious thyroiditis (PT), Injection site abscess (PT), Injection site infection (PT), Instillation site abscess (PT), Instillation site infection (PT), Localised infection (PT), Lymph gland infection (PT), Lymph node abscess (PT), Mediastinitis (PT), Medical device site abscess (PT), Medical device site infection (PT), Mononucleosis syndrome (PT), Mucosal infection (PT), Neonatal infection (PT), Neonatal infective mastitis (PT), Neutropenic infection (PT), Obstetric infection (PT), Omphalitis (PT), Opportunistic infection (PT), Overgrowth of nonsusceptible organisms (PT), Parasite allergy (PT), Parasitic encephalitis (PT), Parasitic oesophagitis (PT), Parathyroid gland abscess (PT), Pathogen resistance (PT), Pelvic abscess (PT), Pelvic infection (PT), Perihepatitis (PT), Peripheral nerve infection (PT), Post

	procedural infection (PT), Postoperative abscess (PT), Postoperative wound infection (PT), Puncture site abscess (PT), Puncture site infection (PT), Purulence (PT), Purulent discharge (PT), Pyuria (PT), Rectovaginal septum abscess (PT), Reproductive tract procedural infection (PT), Respiratory tract infection (PT), Southern tick-associated rash illness (PT), Stitch abscess (PT), Stoma site abscess (PT), Stoma site infection (PT), Superinfection (PT), Systemic infection (PT), Thymus abscess (PT), Thyroglossal cyst infection (PT), Thyroid gland abscess (PT), Tick-borne fever (PT), TORCH infection (PT), Toxic shock syndrome (PT), Transplant abscess (PT), Tropical infectious disease (PT), Vaccination site abscess (PT), Vaccination site infection (PT), Vaccine breakthrough infection (PT), Vascular access site abscess (PT), Vascular access site cellulitis (PT), Vascular access site infection (PT), Vessel puncture site infection (PT), Vestibulitis (PT), Virologic failure (PT), Visceral larva migrans (PT), Wound abscess (PT), Wound infection (PT), Zoonosis (PT), Atypical pneumonia (PT), Bronchitis (PT), Congenital pneumonia (PT), Embolic pneumonia (PT), Haemorrhagic pneumonia (PT), Infectious pleural effusion (PT), Infective exacerbation of bronchiectasis (PT), Infective exacerbation of chronic obstructive airways disease (PT), Infective pulmonary exacerbation of cystic fibrosis (PT), Lower respiratory tract infection (PT), Lung abscess (PT), Mediastinal abscess (PT), Miliary pneumonia (PT), Neonatal pneumonia (PT), Paracancerous pneumonia (PT), Parasitic pneumonia (PT), Pleural infection (PT), Pneumonia (PT), Pneumonia aspiration (PT), Pneumonia necrotising (PT), Post procedural pneumonia (PT), Pyopneumothorax (PT), Sputum purulent (PT), Tracheobronchitis (PT), Young's syndrome (PT), Balanoposthitis infective (PT), Epididymitis (PT), Genital infection male (PT), Hydrocele male infected (PT), Orchitis (PT), Penile abscess (PT), Penile infection (PT), Prostate infection (PT), Prostatic abscess (PT), Pyospermia (PT), Scrotal abscess (PT), Scrotal cellulitis (PT), Scrotal infection (PT), Seminal vesicle abscess (PT), Seminal vesicular infection (PT), Spermatic cord funiculitis (PT), Testicular abscess (PT), Abscess neck (PT), Bezold abscess (PT), Chest wall abscess (PT), Fascial infection (PT), Fournier's gangrene (PT), Infective myositis (PT), Infective tenosynovitis (PT), Muscle abscess (PT), Necrotising fasciitis (PT), Necrotising soft tissue infection (PT), Perineal abscess (PT), Perineal cellulitis (PT), Perineal infection (PT), Psoas abscess (PT), Soft tissue infection (PT), Abdominal sepsis (PT), Amniotic infection syndrome of Blane (PT), Bacteraemia (PT), Bacterial toxaemia (PT), Bacterial translocation (PT), Bacteroides bacteraemia (PT), Cerebral septic infarct (PT), Device related bacteraemia (PT), Device related sepsis (PT), Endotoxaemia (PT), Endotoxic shock (PT), Fungaemia (PT), Intestinal sepsis (PT), Neonatal bacteraemia (PT), Neutropenic sepsis (PT), Pelvic sepsis (PT), Post procedural sepsis (PT), Postpartum sepsis (PT), Pulmonary sepsis (PT), Sepsis (PT), Sepsis neonatal (PT), Sepsis syndrome (PT), Septic cerebral embolism (PT), Septic coagulopathy (PT), Septic embolus (PT), Septic encephalopathy (PT), Septic necrosis (PT), Septic phlebitis (PT), Septic pulmonary embolism (PT), Septic rash (PT), Septic shock (PT), Septic vasculitis (PT), Thrombophlebitis septic (PT), Urosepsis (PT), Viraemia (PT), Wound sepsis (PT), Abscess sweat gland (PT), Acne pustular (PT), Administration site pustule (PT), Application site folliculitis (PT), Application site pustules (PT), Blister infected (PT), Burn infection (PT), Carbuncle (PT), Catheter site pustule (PT), Dermatitis infected (PT), Dermo-hypodermitis (PT), Diabetic foot infection (PT), Ecthyma (PT), Eczema infected (PT), Erysipeloid (PT), Eyelid folliculitis (PT), Impetigo (PT), Implant site pustules (PT), Infected dermal cyst (PT), Infected naevus (PT), Infected skin ulcer (PT), Infusion site pustule (PT), Injection site pustule (PT), Instillation site pustules (PT), Medical device site pustule (PT), Nail bed infection (PT), Nail infection (PT), Periumbilical abscess (PT), Pilonidal cyst congenital (PT), Pilonidal disease (PT), Pitted keratolysis (PT), Pustule (PT), Pyoderma (PT), Radiation site infection (PT), Rash pustular (PT), Sebaceous gland infection (PT), Skin graft infection (PT), Skin infection (PT), Subcutaneous abscess (PT), Subgaleal abscess (PT), Sweat gland infection (PT), Tropical ulcer (PT), Vaccination site pustule (PT), Acute sinusitis (PT), Adenoiditis (PT), Cellulitis laryngeal (PT), Cellulitis pharyngeal (PT), Chronic sinusitis (PT), Chronic tonsillitis (PT), Croup infectious (PT), Epiglottic abscess (PT), Epiglottitis (PT), Epiglottitis obstructive (PT), Laryngitis (PT), Laryngopharyngitis (PT), Laryngotracheitis obstructive (PT), Nasal abscess (PT), Nasal vestibulitis (PT), Nasopharyngitis (PT), Paranasal mucopyocoele (PT), Paranasal sinus abscess (PT), Parapharyngeal space infection (PT), Peritonsillar abscess (PT), Peritonsillitis (PT), Pharyngeal abscess (PT), Pharyngeal pustule (PT), Pharyngitis (PT), Pharyngolaryngeal abscess (PT), Pharyngotonsillitis (PT), Rhinitis (PT), Rhinolaryngitis (PT), Rhinotracheitis (PT), Sinobronchitis (PT), Sinusitis (PT), Subglottic laryngitis (PT), Tonsillitis (PT), Tornwaldt bursitis (PT), Tracheal abscess (PT), Tracheitis (PT), Tracheitis obstructive (PT), Tracheostomy infection (PT), Upper aerodigestive tract infection (PT), Upper respiratory tract infection (PT), , Bladder diverticulitis (PT), Cystitis (PT), Emphysematous cystitis (PT), Emphysematous pyelonephritis (PT), Genitourinary tract infection (PT), Infected urinoma (PT), Kidney infection (PT), Perinephric abscess (PT), Perinephritis (PT), Pyelitis (PT), Pyelocystitis (PT), Pyelonephritis (PT), Pyelonephritis acute (PT), Pyelonephritis chronic (PT), Pyonephrosis (PT), Renal abscess (PT), Renal cyst infection (PT), Renal graft infection (PT), Urachal abscess (PT), Ureter abscess (PT), Ureteritis (PT), Urethral abscess (PT), Urethral carbuncle (PT), Urethral discharge syndrome (PT), Urethral stricture post infection (PT), Urethritis (PT), Urinary bladder abscess (PT), Urinary meatitis (PT), Urinary tract abscess (PT), Urinary tract infection (PT), Urinary tract infection neonatal (PT), Arteriovenous fistula site infection (PT), Arteriovenous graft site abscess (PT), Arteriovenous graft site infection (PT), Arteritis infective (PT), Haematoma infection (PT), Infected lymphocele (PT), Infected varicose vein (PT), Infected vasculitis (PT), Infective aneurysm (PT), Infective aortitis (PT), Infective thrombosis (PT), Infusion site abscess (PT), Infusion site infection (PT), Lymphangitis (PT), Phlebitis infective (PT), Pseudoaneurysm infection (PT), Shunt infection (PT), Vascular device infection (PT), Vascular graft infection (PT), Amoebic brain abscess (PT), Bacterial abscess central nervous system (PT), Brain abscess (PT), Brain empyema (PT), Central nervous system abscess (PT), Dural abscess (PT), Extradural abscess (PT), Fungal abscess central nervous system (PT), Phaeohyphomycotic brain abscess (PT), Spinal cord abscess (PT), Subarachnoid abscess (PT), Subdural abscess (PT), Tuberculous abscess central nervous system (PT), Arachnoiditis (PT), Central nervous system immune reconstitution inflammatory response (PT), Central nervous system inflammation (PT), CNS ventriculitis (PT), Ependymitis (PT), Gliosis (PT), Immune-mediated neurological disorder (PT), Neurosarcoidosis (PT), Vogt-Koyanagi-Harada disease (PT), Bickerstaff's encephalitis (PT), Chronic lymphocytic inflammation with pontine perivascular enhancement responsive to steroids (PT), Encephalitis (PT), Encephalitis allergic (PT), Encephalitis autoimmune (PT), Encephalitis brain stem (PT), Encephalitis haemorrhagic (PT), Encephalitis lethargica (PT), Encephalitis post immunisation (PT), Encephalitis post varicella (PT), Encephalitis toxic (PT), Encephalomyelitis (PT), Immune-mediated encephalitis (PT), Kuru (PT), Limbic encephalitis (PT), Lupus encephalitis (PT), Noninfective encephalitis (PT), Noninfective encephalomyelitis (PT), Paraneoplastic encephalomyelitis (PT), Rasmussen encephalitis (PT), Subacute sclerosing panencephalitis (PT), Balamuthia infection (PT), Cerebral malaria (PT), Cerebral toxoplasmosis (PT), Coccidioides encephalitis (PT), Congenital syphilitic encephalitis (PT), Cryptococcal meningoencephalitis (PT), Encephalitis fungal (PT), Encephalitis meningococcal (PT), Encephalitis protozoal (PT), Encephalitis rickettsial (PT), Encephalomyelitis bacterial (PT), Listeria encephalitis (PT), Meningoencephalitis amoebic (PT), Naegleria infection (PT), Parasitic encephalitis (PT), Adenoviral encephalitis (PT), Adenovirus encephalomyeloradiculitis (PT), Borna virus infection (PT), Encephalitis australia (PT), Encephalitis california (PT), Encephalitis cytomegalovirus (PT), Encephalitis eastern equine (PT), Encephalitis enteroviral (PT), Encephalitis influenzal (PT), Encephalitis Japanese B (PT), Encephalitis mumps (PT), Encephalitis venezuelan equine (PT), Encephalitis viral (PT), Encephalitis western equine (PT), Encephalomyelitis rubella (PT), Encephalomyelitis viral (PT), Herpes simplex encephalitis (PT), Herpes simplex meningoencephalitis (PT), Herpes zoster meningoencephalitis (PT), HIV meningoencephalitis (PT), Human herpesvirus 6 encephalitis (PT), Jamestown Canyon encephalitis (PT), Louping ill (PT), Meningoencephalitis herpes simplex neonatal (PT), Meningoencephalitis herpetic (PT), Meningoencephalitis viral (PT), Murray Valley encephalitis (PT), Polioencephalitis (PT), Rabies (PT), St. Louis encephalitis (PT), Tick-borne viral encephalitis (PT), Varicella encephalitis (PT), West Nile viral infection (PT), Yellow fever vaccine-associated

	neurotropic disease (PT), Congenital syphilitic meningitis (PT), Meningitis bacterial (PT), Meningitis borrelia (PT), Meningitis cronobacter (PT), Meningitis enterococcal (PT), Meningitis Escherichia (PT), Meningitis gonococcal (PT), Meningitis haemophilus (PT), Meningitis leptospiral (PT), Meningitis listeria (PT), Meningitis meningococcal (PT), Meningitis pneumococcal (PT), Meningitis salmonella (PT), Meningitis staphylococcal (PT), Meningitis streptococcal (PT), Meningitis tuberculous (PT), Meningoencephalitis bacterial (PT), Pseudomonas aeruginosa meningitis (PT), Yersinia meningitis (PT), Meningitis aspergillus (PT), Meningitis candida (PT), Meningitis coccidioides (PT), Meningitis cryptococcal (PT), Meningitis exserohilum (PT), Meningitis fungal (PT), Meningitis histoplasma (PT), Adenoviral meningitis (PT), Choriomeningitis lymphocytic (PT), Herpes simplex meningitis (PT), Herpes zoster meningitis (PT), Measles meningitis (PT), Meningitis coxsackie viral (PT), Meningitis echo viral (PT), Meningitis enteroviral (PT), Meningitis herpes (PT), Meningitis mumps (PT), Meningitis viral (PT), Varicella meningitis (PT), Angiostrongylus infection (PT), Chronic infantile neurological cutaneous and articular syndrome (PT), Meningitis (PT), Meningitis aseptic (PT), Meningitis chemical (PT), Meningitis eosinophilic (PT), Meningitis neonatal (PT), Meningitis noninfective (PT), Meningitis toxoplasmal (PT), Meningitis trypanosomal (PT), Pachymeningitis (PT), Rheumatoid meningitis (PT), Acute flaccid myelitis (PT), Acute necrotising myelitis (PT), Bulbar poliomyelitis (PT), Myelitis (PT), Myelitis transverse (PT), Noninfectious myelitis (PT), Paraneoplastic myelopathy (PT), Poliomyelitis (PT), Post polio syndrome (PT), Spinal cord infection (PT), Vaccine associated paralytic poliomyelitis (PT), Viral myelitis (PT), Central nervous system enteroviral infection (PT), Central nervous system fungal infection (PT), Central nervous system infection (PT), Central nervous system viral infection (PT), Cerebral aspergillosis (PT), Cerebral candidiasis (PT), Cerebral fungal infection (PT), Cerebral nocardiosis (PT), Disseminated mucormycosis (PT), Herpes zoster infection neurological (PT), Intracranial infection (PT), Lyme disease (PT), Mucormycosis (PT), Neuroborreliosis (PT), Neurocryptococcosis (PT), Neurocysticercosis (PT), Neurological infection (PT), Neurosyphilis (PT), Panencephalitis (PT), Polyneuropathy mumps (PT), Post viral fatigue syndrome (PT), Rhinocerebral mucormycosis (PT), Rubella infection neurological (PT), Southern tick-associated rash illness (PT), Spinal empyema (PT), Tuberculoma of central nervous system (PT), Tuberculosis of central nervous system (PT)
Drug misuse	Accidental device ingestion (PT narrow), Accidental device ingestion by a child (PT narrow), Accidental exposure to product (PT narrow), Accidental exposure to product by child (PT narrow), Accidental exposure to product by elderly person (PT narrow), Accidental exposure to product packaging (PT narrow), Accidental exposure to product packaging by child (PT narrow), Accidental overdose (PT narrow), Accidental poisoning (PT narrow), Accidental underdose (PT narrow), Accidental use of placebo (PT narrow), Booster dose missed (PT narrow), Circumstance or information capable of leading to device use error (PT narrow), Circumstance or information capable of leading to medication error (PT narrow), Contraindicated device used (PT narrow), Contraindicated product administered (PT narrow), Contraindicated product prescribed (PT narrow), Deprescribing error (PT narrow), Device dispensing error (PT narrow), Device monitoring procedure not performed (PT narrow), Device programming error (PT narrow), Device use confusion (PT narrow), Device use error (PT narrow), Discontinued product administered (PT narrow), Documented hypersensitivity to administered product (PT narrow), Dose calculation error (PT narrow), Drug administered in wrong device (PT narrow), Drug dispensed to wrong patient (PT narrow), Drug dose omission by device (PT narrow), Drug dose titration not performed (PT narrow), Drug monitoring procedure incorrectly performed (PT narrow), Drug monitoring procedure not performed (PT narrow), Drug titration error (PT narrow), Duplicate therapy error (PT narrow), Expired device used (PT narrow), Expired product administered (PT narrow), Exposure via direct contact (PT narrow), Exposure via eye contact (PT narrow), Exposure via skin contact (PT narrow), Extra dose administered (PT narrow), Failure of child resistant product closure (PT narrow), Failure to suspend medication (PT narrow), Inadequate aseptic technique in use of product (PT narrow), Inappropriate schedule of product administration (PT narrow), Inappropriate schedule of product discontinuation (PT narrow), Incomplete course of vaccination (PT narrow), Incorrect disposal of product (PT narrow), Incorrect dosage administered (PT narrow), Incorrect dose administered (PT narrow), Incorrect dose administered by device (PT narrow), Incorrect dose administered by product (PT narrow), Incorrect drug administration rate (PT narrow), Incorrect product administration duration (PT narrow), Incorrect product dosage form administered (PT narrow), Incorrect product formulation administered (PT narrow), Incorrect route of product administration (PT narrow), Intercepted accidental exposure to product by child (PT narrow), Intercepted medication error (PT narrow), Intercepted product administration error (PT narrow), Intercepted product dispensing error (PT narrow), Intercepted product monitoring error (PT narrow), Intercepted product preparation error (PT narrow), Intercepted product prescribing error (PT narrow), Intercepted product selection error (PT narrow), Intercepted product storage error (PT narrow), Intercepted wrong patient selected (PT narrow), Labelled drug-disease interaction medication error (PT narrow), Labelled drug-drug interaction medication error (PT narrow), Labelled drug-food interaction medication error (PT narrow), Labelled drug-genetic interaction medication error (PT narrow), Lack of administration site rotation (PT narrow), Lack of application site rotation (PT narrow), Lack of infusion site rotation (PT narrow), Lack of injection site rotation (PT narrow), Lack of vaccination site rotation (PT narrow), Medical device monitoring error (PT narrow), Medication error (PT narrow), Multiple use of single-use product (PT narrow), Paravenous drug administration (PT narrow), Product administered at inappropriate site (PT narrow), Product administered by wrong person (PT narrow), Product administered to patient of inappropriate age (PT narrow), Product administration error (PT narrow), Product appearance confusion (PT narrow), Product barcode issue (PT narrow), Product confusion (PT narrow), Product design confusion (PT narrow), Product dispensing error (PT narrow), Product dosage form confusion (PT narrow), Product dose confusion (PT narrow), Product dose omission in error (PT narrow), Product label confusion (PT narrow), Product monitoring error (PT narrow), Product name confusion (PT narrow), Product packaging confusion (PT narrow), Product preparation error (PT narrow), Product prescribing error (PT narrow), Product selection error (PT narrow), Product storage error (PT narrow), Product substitution error (PT narrow), Recalled product administered (PT narrow), Single component of a two-component product administered (PT narrow), Therapeutic drug monitoring analysis incorrectly performed (PT narrow), Therapeutic drug monitoring analysis not performed (PT narrow), Transcription medication error (PT narrow), Transfusion with incompatible blood (PT narrow), Unintentional use for unapproved indication (PT narrow), Vaccination error (PT narrow), Wrong device used (PT narrow), Wrong dosage form (PT narrow), Wrong dosage formulation (PT narrow), Wrong dose (PT narrow), Wrong drug (PT narrow), Wrong patient (PT narrow), Wrong patient received product (PT narrow), Wrong product administered (PT narrow), Wrong product procured (PT narrow), Wrong product stored (PT narrow), Wrong rate (PT narrow), Wrong route (PT narrow), Wrong schedule (PT narrow), Wrong strength (PT narrow), Wrong technique in device usage process (PT narrow), Wrong technique in product usage process (PT narrow)
Dementia	Clinical dementia rating scale score abnormal (PT narrow), Corticobasal degeneration (PT narrow), Creutzfeldt-Jakob disease (PT narrow), Dementia (PT narrow), Dementia Alzheimer's type (PT narrow), Dementia of the Alzheimer's type, uncomplicated (PT narrow), Dementia of the Alzheimer's type, with delirium (PT narrow), Dementia of the Alzheimer's type, with delusions (PT narrow), Dementia of the Alzheimer's type, with depressed mood (PT narrow), Dementia with Lewy bodies (PT narrow), Early onset familial Alzheimer's disease (PT narrow), Frontotemporal dementia (PT narrow), Hippocampal atrophy (PT narrow), Hippocampal sclerosis (PT narrow), Korsakoff's syndrome (PT narrow), Mini mental status examination abnormal (PT narrow), Mixed dementia (PT narrow), Presenile dementia (PT narrow), Prion disease (PT narrow), Progressive supranuclear palsy (PT narrow), Scatolia (PT narrow), Senile dementia (PT narrow), Variant Creutzfeldt-Jakob disease (PT narrow), Vascular dementia (PT narrow)

Dehydration	Dehydration (PT narrow), Fluid replacement (PT narrow), Fontanelle depressed (PT narrow), Hypovolaemia (PT narrow), Hypovolaemic shock (PT narrow), Skin turgor decreased (PT narrow)
Hyponatremia (“hyponatraemia”, according to the spelling used in MedDRA),	Antidiuretic hormone abnormality (PT narrow), Blood antidiuretic hormone abnormal (PT narrow), Blood antidiuretic hormone increased (PT narrow), Blood sodium abnormal (PT narrow), Blood sodium decreased (PT narrow), Ectopic antidiuretic hormone secretion (PT narrow), Hyponatraemia (PT narrow), Hyponatraemic coma (PT narrow), Hyponatraemic encephalopathy (PT narrow), Hyponatraemic syndrome (PT narrow), Hypoosmolar state (PT narrow), Inappropriate antidiuretic hormone secretion (PT narrow), Neonatal hyponatraemia (PT narrow), Osmotic demyelination syndrome (PT narrow), Rapid correction of hyponatraemia (PT narrow)
Anticholinergic syndrome	Anticholinergic syndrome (PT)
Hypoglycemia (“hypoglycaemia”, according to the spelling used in MedDRA),	Blood glucose decreased (PT narrow), Glycopenia (PT narrow), Hyperinsulinaemic hypoglycaemia (PT narrow), Hypoglycaemia (PT narrow), Hypoglycaemia neonatal (PT narrow), Hypoglycaemia unawareness (PT narrow), Hypoglycaemic coma (PT narrow), Hypoglycaemic encephalopathy (PT narrow), Hypoglycaemic seizure (PT narrow), Hypoglycaemic unconsciousness (PT narrow), Nesidioblastosis (PT narrow), Neuroglycopenia (PT narrow), Paraneoplastic hypoglycaemia (PT narrow), Postprandial hypoglycaemia (PT narrow), Shock hypoglycaemic (PT narrow)
Seizure disorder	Generalised onset non-motor seizure (PT), Juvenile absence epilepsy (PT), Petit mal epilepsy (PT), Benign familial neonatal convulsions (PT), Generalised tonic-clonic seizure (PT), Automatism epileptic (PT), Dreamy state (PT), Epileptic psychosis (PT), Focal dyscognitive seizures (PT), Temporal lobe epilepsy (PT), Transient epileptic amnesia (PT), Uncinate fits (PT), Autonomic seizure (PT), Deja vu (PT), Epilepsia partialis continua (PT), Parietal lobe epilepsy (PT), Simple partial seizures (PT), Acquired epileptic aphasia (PT), Acute encephalitis with refractory, repetitive partial seizures (PT), Aicardi's syndrome (PT), Alcoholic seizure (PT), Atonic seizures (PT), Atypical benign partial epilepsy (PT), Baltic myoclonic epilepsy (PT), Benign rolandic epilepsy (PT), Biotinidase deficiency (PT), CDKL5 deficiency disorder (PT), CEC syndrome (PT), Change in seizure presentation (PT), Citrate transporter deficiency (PT), Clonic convulsion (PT), Convulsion in childhood (PT), Convulsions local (PT), Convulsive threshold lowered (PT), CSWS syndrome (PT), Drug withdrawal convulsions (PT), Early infantile epileptic encephalopathy with burst-suppression (PT), Eclampsia (PT), Epilepsy (PT), Epilepsy congenital (PT), Epilepsy of infancy with migrating focal seizures (PT), Epilepsy with myoclonic-atonic seizures (PT), Epileptic aura (PT), Epileptic encephalopathy (PT), Eyelid myoclonus (PT), Faciobrachial dystonic seizure (PT), Febrile convulsion (PT), Febrile infection-related epilepsy syndrome (PT), Frontal lobe epilepsy (PT), Gelastic seizure (PT), Hemiconvulsion-hemiplegia-epilepsy syndrome (PT), Hyperglycaemic seizure (PT), Hypocalcaemic seizure (PT), Hypoglycaemic seizure (PT), Hyponatraemic seizure (PT), Idiopathic generalised epilepsy (PT), Idiopathic partial epilepsy (PT), Infantile spasms (PT), Isodicentric chromosome 15 syndrome (PT), Jeavons syndrome (PT), Juvenile myoclonic epilepsy (PT), Lafora's myoclonic epilepsy (PT), Lennox-Gastaut syndrome (PT), Migraine-triggered seizure (PT), Molybdenum cofactor deficiency (PT), Myoclonic epilepsy (PT), Myoclonic epilepsy and ragged-red fibres (PT), Neonatal epileptic seizure (PT), Neonatal seizure (PT), Partial seizures (PT), Partial seizures with secondary generalisation (PT), Phelan-McDermid syndrome (PT), Photosensitive seizure (PT), Post stroke epilepsy (PT), Post stroke seizure (PT), Postictal depression (PT), Postictal psychosis (PT), Post-traumatic epilepsy (PT), Progressive encephalopathy, hypsarrhythmia and optic atrophy syndrome (PT), Psychogenic seizure (PT), PURA syndrome (PT), Seizure (PT), Seizure anoxic (PT), Seizure cluster (PT), Seizure like phenomena (PT), Severe myoclonic epilepsy of infancy (PT), Sleep related hypermotor epilepsy (PT), Status epilepticus (PT), Sudden unexplained death in epilepsy (PT), Tonic clonic movements (PT), Tonic convulsion (PT), Tonic posturing (PT), Tuberous sclerosis complex (PT)
Drug abuse, dependence, withdrawal	Caffeine dependence (PT narrow), Dopamine dysregulation syndrome (PT narrow), Drug abuse (PT narrow), Drug abuser (PT narrow), Drug dependence (PT narrow), Drug dependence, antepartum (PT narrow), Drug dependence, postpartum (PT narrow), Drug use disorder (PT narrow), Drug use disorder, antepartum (PT narrow), Drug use disorder, postpartum (PT narrow), Intentional device misuse (PT narrow), Intentional overdose (PT narrow), Intentional product misuse (PT narrow), Maternal use of illicit drugs (PT narrow), Neonatal complications of substance abuse (PT narrow), Substance abuse (PT narrow), Substance abuser (PT narrow), Substance dependence (PT narrow), Substance use disorder (PT narrow), Drug withdrawal convulsions (PT narrow), Drug withdrawal headache (PT narrow), Drug withdrawal maintenance therapy (PT narrow), Drug withdrawal syndrome (PT narrow), Drug withdrawal syndrome neonatal (PT narrow)
Central nervous system vascular disorders	Balint's syndrome (PT narrow), Basilar artery aneurysm (PT narrow), Carotid artery aneurysm (PT narrow), Carotid artery dissection (PT narrow), Cerebral cavernous malformation (PT narrow), Cerebral endovascular aneurysm repair (PT narrow), Cerebral reperfusion injury (PT narrow), Cerebral ventricular rupture (PT narrow), Cerebrovascular accident prophylaxis (PT narrow), Cerebrovascular pseudoaneurysm (PT narrow), Charcot-Bouchard microaneurysms (PT narrow), Congenital hemiparesis (PT narrow), CSF bilirubin positive (PT narrow), Delayed ischaemic neurological deficit (PT narrow), Hemianaesthesia (PT narrow), Hemiasomatognosia (PT narrow), Hemiataxia (PT narrow), Hemidysaesthesia (PT narrow), Hemihyperaesthesia (PT narrow), Hemihypoaesthesia (PT narrow), Hemiparaesthesia (PT narrow), Hemiparesis (PT narrow), Hemiplegia (PT narrow), Intra-cerebral aneurysm operation (PT narrow), Intracranial aneurysm (PT narrow), Lateropulsion (PT narrow), Malignant middle cerebral artery syndrome (PT narrow), Metabolic stroke (PT narrow), Post stroke depression (PT narrow), Posthaemorrhagic hydrocephalus (PT narrow), Precerebral artery aneurysm (PT narrow), Precerebral artery dissection (PT narrow), Septic cerebral embolism (PT narrow), Vein of Galen aneurysmal malformation (PT narrow), Vertebral artery aneurysm (PT narrow), Vertebral artery dissection (PT narrow), Basal ganglia haematoma (PT narrow), Basal ganglia haemorrhage (PT narrow), Basal ganglia stroke (PT narrow), Basilar artery perforation (PT narrow), Brain stem haematoma (PT narrow), Brain stem haemorrhage (PT narrow), Brain stem microhaemorrhage (PT narrow), Brain stem stroke (PT narrow), Carotid aneurysm rupture (PT narrow), Carotid artery perforation (PT narrow), Central nervous system haemorrhage (PT narrow), Cerebellar haematoma (PT narrow), Cerebellar haemorrhage (PT narrow), Cerebellar microhaemorrhage (PT narrow), Cerebellar stroke (PT narrow), Cerebral aneurysm perforation (PT narrow), Cerebral aneurysm ruptured syphilitic (PT narrow), Cerebral arteriovenous malformation haemorrhagic (PT narrow), Cerebral artery perforation (PT narrow), Cerebral cyst haemorrhage (PT narrow), Cerebral haematoma (PT narrow), Cerebral haemorrhage (PT narrow), Cerebral haemorrhage foetal (PT narrow), Cerebral haemorrhage neonatal (PT narrow), Cerebral microhaemorrhage (PT narrow), Cerebrovascular accident (PT narrow), Cerebrovascular disorder (PT narrow), Epidural haemorrhage (PT narrow), Extra-axial haemorrhage (PT narrow), Extradural haematoma (PT narrow), Extradural haematoma evacuation (PT narrow), Extraischaemic cerebral haematoma (PT narrow), Foville syndrome (PT narrow), Haemorrhage intracranial (PT narrow), Haemorrhagic cerebellar infarction (PT narrow), Haemorrhagic cerebral infarction (PT narrow), Haemorrhagic stroke (PT narrow), Haemorrhagic transformation stroke (PT narrow), Intracerebral haematoma evacuation (PT narrow), Intracranial haematoma (PT narrow), Intracranial haemorrhage neonatal (PT narrow), Intracranial tumour haemorrhage (PT narrow), Intraventricular haemorrhage (PT narrow), Intraventricular haemorrhage neonatal (PT narrow), Meningorrhagia (PT narrow), Perinatal stroke (PT narrow), Periventricular haemorrhage neonatal (PT narrow), Pituitary apoplexy (PT narrow), Pituitary haemorrhage (PT narrow), Putamen haemorrhage (PT narrow), Ruptured cerebral aneurysm (PT narrow), Spinal

	cord haematoma (PT narrow), Spinal cord haemorrhage (PT narrow), Spinal epidural haematoma (PT narrow), Spinal epidural haemorrhage (PT narrow), Spinal stroke (PT narrow), Spinal subarachnoid haemorrhage (PT narrow), Spinal subdural haematoma (PT narrow), Spinal subdural haemorrhage (PT narrow), Stroke in evolution (PT narrow), Subarachnoid haematoma (PT narrow), Subarachnoid haemorrhage (PT narrow), Subarachnoid haemorrhage neonatal (PT narrow), Subdural haematoma (PT narrow), Subdural haematoma evacuation (PT narrow), Subdural haemorrhage (PT narrow), Subdural haemorrhage neonatal (PT narrow), Thalamus haemorrhage (PT narrow), Vertebral artery perforation (PT narrow), Vertebrobasilar stroke (PT narrow), Amaurosis fugax (PT narrow), Basal ganglia infarction (PT narrow), Basal ganglia stroke (PT narrow), Basilar artery occlusion (PT narrow), Basilar artery stenosis (PT narrow), Basilar artery thrombosis (PT narrow), Benedikt's syndrome (PT narrow), Brachiocephalic arteriosclerosis (PT narrow), Brachiocephalic artery occlusion (PT narrow), Brachiocephalic artery stenosis (PT narrow), Brain hypoxia (PT narrow), Brain stem embolism (PT narrow), Brain stem infarction (PT narrow), Brain stem ischaemia (PT narrow), Brain stem stroke (PT narrow), Brain stem thrombosis (PT narrow), Brain stent insertion (PT narrow), CADASIL (PT narrow), Capsular warning syndrome (PT narrow), CARASIL syndrome (PT narrow), Carotid angioplasty (PT narrow), Carotid arterial embolus (PT narrow), Carotid arteriosclerosis (PT narrow), Carotid artery bypass (PT narrow), Carotid artery disease (PT narrow), Carotid artery insufficiency (PT narrow), Carotid artery occlusion (PT narrow), Carotid artery restenosis (PT narrow), Carotid artery stenosis (PT narrow), Carotid artery stent insertion (PT narrow), Carotid artery stent removal (PT narrow), Carotid artery thrombosis (PT narrow), Carotid endarterectomy (PT narrow), Carotid revascularisation (PT narrow), Cerebellar artery occlusion (PT narrow), Cerebellar artery thrombosis (PT narrow), Cerebellar atherosclerosis (PT narrow), Cerebellar embolism (PT narrow), Cerebellar infarction (PT narrow), Cerebellar ischaemia (PT narrow), Cerebellar stroke (PT narrow), Cerebral arteriosclerosis (PT narrow), Cerebral artery embolism (PT narrow), Cerebral artery occlusion (PT narrow), Cerebral artery restenosis (PT narrow), Cerebral artery stenosis (PT narrow), Cerebral artery stent insertion (PT narrow), Cerebral artery thrombosis (PT narrow), Cerebral gas embolism (PT narrow), Cerebral infarction (PT narrow), Cerebral infarction foetal (PT narrow), Cerebral ischaemia (PT narrow), Cerebral microembolism (PT narrow), Cerebral microinfarction (PT narrow), Cerebral revascularisation (PT narrow), Cerebral septic infarct (PT narrow), Cerebral small vessel ischaemic disease (PT narrow), Cerebral thrombosis (PT narrow), Cerebral vascular occlusion (PT narrow), Cerebral vasoconstriction (PT narrow), Cerebral venous thrombosis (PT narrow), Cerebrovascular accident (PT narrow), Cerebrovascular disorder (PT narrow), Cerebrovascular insufficiency (PT narrow), Cerebrovascular stenosis (PT narrow), Claude's syndrome (PT narrow), Delayed ischaemic neurological deficit (PT narrow), Embolic cerebellar infarction (PT narrow), Embolic cerebral infarction (PT narrow), Embolic stroke (PT narrow), Foville syndrome (PT narrow), Hypoxic-ischaemic encephalopathy (PT narrow), Inner ear infarction (PT narrow), Internal capsule infarction (PT narrow), Ischaemic cerebral infarction (PT narrow), Ischaemic stroke (PT narrow), Lacunar infarction (PT narrow), Lacunar stroke (PT narrow), Lateral medullary syndrome (PT narrow), Migrainous infarction (PT narrow), Millard-Gubler syndrome (PT narrow), Moyamoya disease (PT narrow), Perinatal stroke (PT narrow), Post cardiac arrest syndrome (PT narrow), Post procedural stroke (PT narrow), Precerebral arteriosclerosis (PT narrow), Precerebral artery embolism (PT narrow), Precerebral artery occlusion (PT narrow), Precerebral artery thrombosis (PT narrow), Pseudo-occlusion of internal carotid artery (PT narrow), Reversible cerebral vasoconstriction syndrome (PT narrow), Reversible ischaemic neurological deficit (PT narrow), Spinal artery embolism (PT narrow), Spinal artery thrombosis (PT narrow), Spinal cord infarction (PT narrow), Spinal cord ischaemia (PT narrow), Spinal stroke (PT narrow), Stroke in evolution (PT narrow), Subclavian steal syndrome (PT narrow), Thalamic infarction (PT narrow), Thrombotic cerebral infarction (PT narrow), Thrombotic stroke (PT narrow), Transient ischaemic attack (PT narrow), Vascular encephalopathy (PT narrow), Vascular stent occlusion (PT narrow), Vascular stent stenosis (PT narrow), Vertebral artery arteriosclerosis (PT narrow), Vertebral artery occlusion (PT narrow), Vertebral artery stenosis (PT narrow), Vertebral artery thrombosis (PT narrow), Vertebrobasilar insufficiency (PT narrow), Vertebrobasilar stroke (PT narrow), Weber's syndrome (PT narrow), Central nervous system vasculitis (PT narrow), Cerebral arteritis (PT narrow), Cerebral capillary telangiectasia (PT narrow), Cerebral circulatory failure (PT narrow), Cerebral congestion (PT narrow), Cerebral hypoperfusion (PT narrow), Cerebral venous sinus thrombosis (PT narrow), Chronic cerebrospinal venous insufficiency (PT narrow), Dural arteriovenous fistula (PT narrow), Sigmoid sinus thrombosis (PT narrow), Superior sagittal sinus thrombosis (PT narrow), Transverse sinus thrombosis (PT narrow)
Hearing impairment	Acoustic stimulation tests abnormal (PT narrow), Altered pitch perception (PT narrow), Audiogram abnormal (PT narrow), Auditory disorder (PT narrow), Auditory recruitment (PT narrow), Autophony (PT narrow), Barotitis media (PT narrow), Bone anchored hearing aid implantation (PT narrow), Cochlea implant (PT narrow), Conductive deafness (PT narrow), Deafness (PT narrow), Deafness bilateral (PT narrow), Deafness neurosensory (PT narrow), Deafness occupational (PT narrow), Deafness permanent (PT narrow), Deafness transitory (PT narrow), Deafness unilateral (PT narrow), Diplacusis (PT narrow), Dysacusis (PT narrow), Electrocochleogram abnormal (PT narrow), Eustachian tube disorder (PT narrow), Eustachian tube dysfunction (PT narrow), Eustachian tube obstruction (PT narrow), Haematotympanum (PT narrow), Hearing aid therapy (PT narrow), Hearing therapy (PT narrow), Hyperacusis (PT narrow), Hypoacusis (PT narrow), Middle ear adhesions (PT narrow), Middle ear effusion (PT narrow), Middle ear inflammation (PT narrow), Misophonia (PT narrow), Mixed deafness (PT narrow), Neonatal deafness (PT narrow), Neonatal hypoacusis (PT narrow), Neurosensory hypoacusis (PT narrow), Noninfective myringitis (PT narrow), Ossicle disorder (PT narrow), Otoacoustic emissions test abnormal (PT narrow), Otosclerosis (PT narrow), Ototoxicity (PT narrow), Paracusis (PT narrow), Presbyacusis (PT narrow), Rinne tuning fork test abnormal (PT narrow), Sudden hearing loss (PT narrow), Tinnitus (PT narrow), Tinnitus retraining therapy (PT narrow), Tympanic membrane atrophic (PT narrow), Tympanic membrane disorder (PT narrow), Tympanic membrane perforation (PT narrow), Tympanic membrane scarring (PT narrow), Tympanometry abnormal (PT narrow), Tympanosclerosis (PT narrow), Weber tuning fork test abnormal (PT narrow)
Visual impairment	Acquired hypertrophy of the retinal pigment epithelium (PT narrow), Acquired pigmented retinopathy (PT narrow), Acute macular neuroretinopathy (PT narrow), Age-related macular degeneration (PT narrow), AIDS retinopathy (PT narrow), Amaurosis fugax (PT narrow), Anaemic retinopathy (PT narrow), Angiogram retina abnormal (PT narrow), Arteriosclerotic retinopathy (PT narrow), Autoimmune retinopathy (PT narrow), Benign neoplasm of retina (PT narrow), Biopsy retina abnormal (PT narrow), Birdshot chorioretinopathy (PT narrow), Central serous chorioretinopathy (PT narrow), Cholesterolosis bulbi (PT narrow), Chorioretinal disorder (PT narrow), Chorioretinal scar (PT narrow), Chorioretinitis (PT narrow), Chorioretinopathy (PT narrow), Choroidal osteoma (PT narrow), Colour blindness (PT narrow), Colour blindness acquired (PT narrow), Colour vision tests abnormal (PT narrow), Colour vision tests abnormal blue-yellow (PT narrow), Colour vision tests abnormal red-green (PT narrow), Commotio retinae (PT narrow), Cystoid macular oedema (PT narrow), Delayed light adaptation (PT narrow), Detachment of macular retinal pigment epithelium (PT narrow), Detachment of retinal pigment epithelium (PT narrow), Diabetic retinal oedema (PT narrow), Diabetic retinopathy (PT narrow), Diffuse uveal melanocytic proliferation (PT narrow), Disruption of the photoreceptor inner segment-outer segment (PT narrow), Dry age-related macular degeneration (PT narrow), Epiretinal membrane (PT narrow), Epiretinal membrane peel (PT narrow), Extraocular retinoblastoma (PT narrow), Exudative retinopathy (PT narrow), Eye naevus (PT narrow), Foveal degeneration (PT narrow), Fundoscopy abnormal (PT narrow), Haemorrhagic occlusive retinal vasculitis (PT narrow), Hypotony maculopathy (PT narrow), Immune

	recovery uveitis (PT narrow), Internal limiting membrane peeling (PT narrow), IRVAN syndrome (PT narrow), Laser floater treatment (PT narrow), Leukaemic retinopathy (PT narrow), Lipaemia retinalis (PT narrow), Macular cherry-red spots (PT narrow), Macular cyst (PT narrow), Macular degeneration (PT narrow), Macular detachment (PT narrow), Macular fibrosis (PT narrow), Macular hole (PT narrow), Macular ischaemia (PT narrow), Macular oedema (PT narrow), Macular opacity (PT narrow), Macular pigmentation (PT narrow), Macular pseudohole (PT narrow), Macular reflex abnormal (PT narrow), Macular rupture (PT narrow), Macular scar (PT narrow), Macular telangiectasia (PT narrow), Macular thickening (PT narrow), Maculopathy (PT narrow), Malignant neoplasm of retina (PT narrow), Metamorphopsia (PT narrow), Myopic chorioretinal degeneration (PT narrow), Myopic traction maculopathy (PT narrow), Necrotising retinitis (PT narrow), Neovascular age-related macular degeneration (PT narrow), Neuropathy, ataxia, retinitis pigmentosa syndrome (PT narrow), Noninfective chorioretinitis (PT narrow), Noninfective retinitis (PT narrow), Non-proliferative retinopathy (PT narrow), Paraneoplastic retinopathy (PT narrow), Peripapillary pigmentation (PT narrow), Peripheral exudative haemorrhagic chorioretinopathy (PT narrow), Photopsia (PT narrow), Post thrombotic retinopathy (PT narrow), Pupillary light reflex tests abnormal (PT narrow), Purtscher retinopathy (PT narrow), Radiation retinopathy (PT narrow), Retinal aneurysm (PT narrow), Retinal aneurysm rupture (PT narrow), Retinal arteriovenous malformation (PT narrow), Retinal artery embolism (PT narrow), Retinal artery occlusion (PT narrow), Retinal artery spasm (PT narrow), Retinal artery stenosis (PT narrow), Retinal artery thrombosis (PT narrow), Retinal collateral vessels (PT narrow), Retinal coloboma (PT narrow), Retinal cryoablation (PT narrow), Retinal cyst (PT narrow), Retinal cyst excision (PT narrow), Retinal degeneration (PT narrow), Retinal depigmentation (PT narrow), Retinal deposits (PT narrow), Retinal detachment (PT narrow), Retinal disorder (PT narrow), Retinal drusen (PT narrow), Retinal dystrophy (PT narrow), Retinal exudates (PT narrow), Retinal fibrosis (PT narrow), Retinal fovea disorder (PT narrow), Retinal function test abnormal (PT narrow), Retinal haemorrhage (PT narrow), Retinal implant (PT narrow), Retinal infarction (PT narrow), Retinal infiltrates (PT narrow), Retinal injury (PT narrow), Retinal ischaemia (PT narrow), Retinal laser coagulation (PT narrow), Retinal melanocytoma (PT narrow), Retinal melanoma (PT narrow), Retinal microangiopathy (PT narrow), Retinal migraine (PT narrow), Retinal neoplasm (PT narrow), Retinal neovascularisation (PT narrow), Retinal occlusive vasculitis (PT narrow), Retinal oedema (PT narrow), Retinal operation (PT narrow), Retinal pallor (PT narrow), Retinal perivascular sheathing (PT narrow), Retinal phototoxicity (PT narrow), Retinal pigment epithelial tear (PT narrow), Retinal pigment epitheliopathy (PT narrow), Retinal pigmentation (PT narrow), Retinal scar (PT narrow), Retinal tear (PT narrow), Retinal telangiectasia (PT narrow), Retinal thickening (PT narrow), Retinal toxicity (PT narrow), Retinal transplant (PT narrow), Retinal tumour excision (PT narrow), Retinal vascular disorder (PT narrow), Retinal vascular occlusion (PT narrow), Retinal vascular thrombosis (PT narrow), Retinal vasculitis (PT narrow), Retinal vein occlusion (PT narrow), Retinal vein thrombosis (PT narrow), Retinal vein varices (PT narrow), Retinal vessel avulsion (PT narrow), Retinal white dots syndrome (PT narrow), Retinal white without pressure (PT narrow), Retinitis (PT narrow), Retinitis pigmentosa (PT narrow), Retinoblastoma (PT narrow), Retinogram abnormal (PT narrow), Retinopathy (PT narrow), Retinopathy haemorrhagic (PT narrow), Retinopathy hypertensive (PT narrow), Retinopathy hyperviscosity (PT narrow), Retinopathy of prematurity (PT narrow), Retinopathy proliferative (PT narrow), Retinopathy sickle cell (PT narrow), Retinopathy solar (PT narrow), Retinopexy (PT narrow), Retinoschisis (PT narrow), Rhegmatogenous retinal detachment (PT narrow), Scintillating scotoma (PT narrow), Scleral buckling surgery (PT narrow), Serous retinal detachment (PT narrow), Serous retinopathy (PT narrow), Serpiginous choroiditis (PT narrow), Subretinal fibrosis (PT narrow), Subretinal fluid (PT narrow), Subretinal haematoma (PT narrow), Subretinal hyperreflective exudation (PT narrow), Tractional retinal detachment (PT narrow), Transpupillary thermotherapy (PT narrow), Tunnel vision (PT narrow), Venous stasis retinopathy (PT narrow), Visual field tests abnormal (PT narrow), Vitreal cells (PT narrow), Vitrectomy (PT narrow), Vitreomacular interface abnormal (PT narrow), Vitreoretinal traction syndrome (PT narrow), Vitreous adhesions (PT narrow), Vitreous detachment (PT narrow), Vitreous disorder (PT narrow), Vitreous floaters (PT narrow), Vitreous haematoma (PT narrow), Vitreous haemorrhage (PT narrow), Vitreous haze (PT narrow), Vitritis (PT narrow), Benign neoplasm of optic nerve (PT narrow), Glaucomatous optic disc atrophy (PT narrow), Myelin oligodendrocyte glycoprotein antibody-associated disease (PT narrow), Neuromyelitis optica pseudo relapse (PT narrow), Neuromyelitis optica spectrum disorder (PT narrow), Optic atrophy (PT narrow), Optic disc disorder (PT narrow), Optic disc drusen (PT narrow), Optic disc haemorrhage (PT narrow), Optic disc hyperaemia (PT narrow), Optic disc pigmentation (PT narrow), Optic disc telangiectasia (PT narrow), Optic disc traction syndrome (PT narrow), Optic disc vascular disorder (PT narrow), Optic discs blurred (PT narrow), Optic glioma (PT narrow), Optic ischaemic neuropathy (PT narrow), Optic nerve compression (PT narrow), Optic nerve cup/disc ratio decreased (PT narrow), Optic nerve cup/disc ratio increased (PT narrow), Optic nerve cupping (PT narrow), Optic nerve disorder (PT narrow), Optic nerve infarction (PT narrow), Optic nerve injury (PT narrow), Optic nerve neoplasm (PT narrow), Optic nerve operation (PT narrow), Optic nerve sheath haemorrhage (PT narrow), Optic neuritis (PT narrow), Optic neuritis meningococcal (PT narrow), Optic neuropathy (PT narrow), Optic perineuritis (PT narrow), Orbital apex syndrome (PT narrow), Papillitis (PT narrow), Papilloedema (PT narrow), Progressive relapsing multiple sclerosis (PT narrow), Pseudopapilloedema (PT narrow), Subacute myelo-opticoneuropathy (PT narrow), Toxic optic neuropathy (PT narrow), Visual evoked potentials abnormal (PT narrow), Acquired lenticonus (PT narrow), Acute myopia (PT narrow), Anterior capsular rupture (PT narrow), Aphakia (PT narrow), Aphakic glaucoma (PT narrow), Atopic cataract (PT narrow), Bioptic eye surgery (PT narrow), Cataract (PT narrow), Cataract cortical (PT narrow), Cataract diabetic (PT narrow), Cataract nuclear (PT narrow), Cataract operation (PT narrow), Cataract operation complication (PT narrow), Cataract subcapsular (PT narrow), Colour blindness acquired (PT narrow), Dysphotopsia (PT narrow), Fibrin deposition on lens postoperative (PT narrow), Floppy iris syndrome (PT narrow), Hepato-lenticular degeneration (PT narrow), Lens capsulotomy (PT narrow), Lens density increased (PT narrow), Lens discolouration (PT narrow), Lens feathering (PT narrow), Lenticular opacities (PT narrow), Lenticular pigmentation (PT narrow), Phacocystectomy (PT narrow), Posterior capsule neovascularisation (PT narrow), Posterior capsule opacification (PT narrow), Posterior capsule rupture (PT narrow), Posterior lens capsulotomy (PT narrow), Pseudophakic bullous keratopathy (PT narrow), Radiation cataract (PT narrow), Suture fixation of intraocular lens (PT narrow), Toxic anterior segment syndrome (PT narrow), Toxic cataract (PT narrow), Acute myopia (PT narrow), Angle closure glaucoma (PT narrow), Aphakic glaucoma (PT narrow), Bleb revision (PT narrow), Borderline glaucoma (PT narrow), Developmental glaucoma (PT narrow), Diabetic glaucoma (PT narrow), Episcleral venous pressure increased (PT narrow), Exfoliation glaucoma (PT narrow), Fundoscopy abnormal (PT narrow), Glaucoma (PT narrow), Glaucoma drainage device placement (PT narrow), Glaucoma drug therapy (PT narrow), Glaucoma surgery (PT narrow), Glaucoma traumatic (PT narrow), Glaucomatocyclitic crises (PT narrow), Glaucomatous optic disc atrophy (PT narrow), Gonioscopy abnormal (PT narrow), Halo vision (PT narrow), Idiopathic dilated episcleral vessels (PT narrow), Intraocular pressure fluctuation (PT narrow), Intraocular pressure increased (PT narrow), Intraocular pressure test abnormal (PT narrow), Iridotomy (PT narrow), Laser suture lysis after trabeculectomy (PT narrow), Loss of visual contrast sensitivity (PT narrow), Malignant glaucoma (PT narrow), Normal tension glaucoma (PT narrow), Ocular hypertension (PT narrow), Open angle glaucoma (PT narrow), Ophthalmic fluid drainage (PT narrow), Optic discs blurred (PT narrow), Optic nerve cup/disc ratio increased (PT narrow), Optic nerve cupping (PT narrow), Phacolytic glaucoma (PT narrow), Phacotrabeculectomy (PT narrow), Pigmentary glaucoma (PT narrow), Pseudophakic glaucoma (PT narrow), Pupillary light reflex tests abnormal (PT narrow), Slit-lamp tests abnormal (PT narrow), Trabecular aspiration (PT narrow), Trabeculectomy (PT narrow), Trabeculoplasty (PT narrow), Uveitic glaucoma (PT narrow), Uveitis-glaucoma-hyphaema syndrome (PT narrow), Viscocanalostomy (PT narrow), Visual field tests abnormal (PT narrow)

Supplementary Table 3 - Colinearity analysis, based on a multivariate analysis of the association between SSRI use and reports of delirium

		Degrees of freedom	Generalized variance-inflation factors
**Sex**		1	1.00
**Age**		1	1.00
**Geographic region**		5	1.03
**Potentially associated illnesses known to induce delirium**	Constipation	1	1.00
	Acute urine retention	1	1.00
	Alcohol	1	1.00
	Infections unspecified	1	1.02
	Drug misuse	1	1.01
	Dementia	1	1.01
	Dehydration	1	1.01
	Hyponatremia	1	1.02
	Anticholinergic syndrome	1	1.00
	Hypoglycemia	1	1.00
	Seizure disorder	1	1.01
	Drug abuse, dependence, withdrawal	1	1.00
	Central nervous system vascular disorders	1	1.01
	Hearing impairment	1	1.00
	Visual impairment	1	1.00
**Potentially associated prescriptions of drugs known to induce delirium**	Opioids	1	1.12
	Antipsychotics	1	1.05
	Anxiolytics	1	1.27
	Hypnotics	1	1.05
**Antidepressant class**	Selective serotonin reuptake inhibitors (SSRIs)	1	1.16

Supplementary Table 4 - The risk of concomitant delirium and hyponatremia in older adults, and characteristics of the corresponding population

A)

		Subgroup analysis for the co-event delirium-hyponatremia			
		Number of delirium-hyponatremia reports	Number of all reports	Univariate analysis r-OR[95%CI]	Multivariate analysis r-OR[95%CI]
**Total number of reports for 65+ age group**		3532	6048742		
**Antidepressant classes**	Non-selective monoamine reuptake inhibitors	143	55646	4.55[3.85-5.38]	1.03[0.87-1.23]
	Selective serotonin reuptake inhibitors	857	148194	12.82[11.87-13.85]	4.46[4.01-4.96]
	Serotonin-norepinephrine reuptake inhibitors	200	54171	6.66[5.78-7.69]	1.25[1.07-1.46]
	Monoamine oxidase inhibitors	9	2256	6.87[3.57-13.23]	3.09[1.60-5.99]
	Alpha-2-adrenergic receptor antagonists	111	31470	6.22[5.15-7.52]	1.72[1.41-2.09]
	Other antidepressants	397	133197	5.64[5.08-6.26]	1.47[1.30-1.65]

B)

		Variables introduced into the multivariate analysis*		
		Number of delirium-hyponatremia reports	Number of all reports	Univariate analysis r-OR[95%CI]
**Sex****	Men	1015	2552052	0.55[0.51-0.59]
	Women	2485	3427187	Ref
**Age**	65-74	1046	3401357	Ref
	75+	2486	2647385	3.06[2.84-3.28]
**Potentially associated illnesses known to induce delirium**	Constipation	75	66587	1.95[1.55-2.45]
	Acute urine retention	47	12283	6.65[4.98-8.87]
	Alcohol	2	327	10.54[2.62-42.33]
	Infections unspecified	313	308170	1.81[1.61-2.04]
	Drug misuse	57	191303	0.50[0.39-0.65]
	Dementia	39	10994	6.15[4.48-8.44]
	Dehydration	250	34166	13.50[11.87-15.36]
	Anticholinergic syndrome	1	162	10.63[1.49-75.97]
	Hypoglycemia	33	45506	1.24[0.88-1.75]
	Seizure disorder	178	34392	9.32[8.02-10.85]
	Drug abuse, dependence, withdrawal	16	29000	0.94[0.58-1.54]
	Central nervous system vascular disorders	116	141280	1.42[1.18-1.71]
	Hearing impairment	20	37396	0.92[0.59-1.42]
	Visual impairment	25	43516	0.98[0.66-1.46]
**Potentially associated prescriptions of drugs known to induce delirium**	Opioids	1516	1175087	3.12[2.92-3.33]
	Antipsychotics	406	173605	4.40[3.97-4.88]
	Anxiolytics	1208	415752	7.06[6.59-7.57]
	Hypnotics	352	181304	3.59[3.21-4.01]

*** including geographic region**
**** 32 NA (1% missing data for sex variable)**

Supplementary Table 5- The risk of delirium in the 65-74 age group, and the characteristics of the corresponding population

A)

		Subgroup analysis of the 65-74 age group			
		Number of delirium reports	Number of all reports	Univariate analysis r-OR[95%CI]	Multivariate analysis r-OR[95%CI]
**Total number of reports for 65-74 age group**		36155	3401357		
**Antidepressant classes**	Non-selective monoamine reuptake inhibitors	1172	32342	3.58[3.38-3.80]	1.31[1.23-1.40]
	Selective serotonin reuptake inhibitors	2436	76193	3.22[3.09-3.36]	1.06[1.01-1.11]
	Serotonin-norepinephrine reuptake inhibitors	1082	32792	3.24[3.05-3.45]	0.91[0.85-0.97]
	Monoamine oxidase inhibitors	82	1340	6.08[4.86-7.60]	3.00[2.38-3.79]
	Alpha-2-adrenergic receptor antagonists	507	13137	3.78[3.45-4.13]	1.34[1.22-1.48]
	Other antidepressants	2543	73503	3.51[3.37-3.66]	1.22[1.17-1.28]

B)

		Variables introduced into the multivariate analysis*		
		Number of delirium reports	Number of all reports	Univariate analysis r-OR[95%CI]
**Sex****	Men	17578	1458171	1.26[1.23-1.29]
	Women	18265	1904840	Ref
**Potentially associated illnesses known to induce delirium**	Constipation	716	36284	1.89[1.76-2.04]
	Acute urine retention	210	5682	3.59[3.12-4.12]
	Alcohol	18	258	6.98[4.33-11.28]
	Infections unspecified	3017	181071	1.63[1.57-1.69]
	Drug misuse	1120	102155	1.03[0.97-1.10]
	Dementia	422	3242	14.08[12.71-15.61]
	Dehydration	856	17259	4.95[4.62-5.31]
	Hyponatremia	1046	14733	7.30[6.84-7.78]
	Anticholinergic syndrome	20	82	30.04[18.15-49.73]
	Hypoglycemia	704	22807	3.00[2.78-3.24]
	Seizure disorder	1036	18188	5.76[5.40-6.14]
	Drug abuse, dependence, withdrawal	473	17120	2.67[2.43-2.92]
	Central nervous system vascular disorders	1506	59287	2.49[2.36-2.62]
	Hearing impairment	469	21607	2.08[1.90-2.28]
	Visual impairment	263	24258	1.02[0.90-1.15]
**Potentially associated prescriptions of drugs known to induce delirium**	Opioids	11776	616594	2.21[2.16-2.26]
	Antipsychotics	4169	89719	5.00[4.83-5.16]
	Anxiolytics	6983	223937	3.47[3.38-3.57]
	Hypnotics	3744	93280	4.23[4.08-4.37]

*** including geographic region**
**** 312 NA (1% missing data for sex variable)**

Supplementary Table 6 - The risk of delirium in the 75+ age group, and the characteristics of the corresponding population

A)

		Subgroup analysis of the 75+ age group			
		Number of delirium reports	Number of all reports	Univariate analysis r-OR[95%CI]	Multivariate analysis r-OR[95%CI]
**Total number of reports for 75+ age group**		51369	2647385		
**Antidepressant classes**	Non-selective monoamine reuptake inhibitors	1486	23304	3.51[3.33-3.71]	1.42[1.34-1.51]
	Selective serotonin reuptake inhibitors	4039	72001	3.17[3.07-3.28]	1.20[1.16-1.25]
	Serotonin-norepinephrine reuptake inhibitors	1165	21379	2.96[2.79-3.14]	0.86[0.81-0.92]
	Monoamine oxidase inhibitors	76	916	4.58[3.62-5.79]	2.49[1.95-3.18]
	Alpha-2-adrenergic receptor antagonists	1046	18333	3.10[2.91-3.30]	1.34[1.22-1.48]
	Other antidepressants	3307	59694	3.10[2.99-3.21]	1.22[1.17-1.28]

B)

		Variables introduced into the multivariate analysis*		
		Number of delirium reports	Number of all reports	Univariate analysis r-OR[95%CI]
**Sex****	Men	22077	1093881	1.07[1.05-1.09]
	Women	28858	1522347	Ref
**Potentially associated illnesses known to induce delirium**	Constipation	910	30303	1.57[1.47-1.68]
	Acute urine retention	433	6601	3.57[3.24-3.94]
	Alcohol	8	69	6.63[3.17-13.85]
	Infections unspecified	3425	127099	1.43[1.38-1.48]
	Drug misuse	1521	89148	0.87[0.83-0.92]
	Dementia	896	7752	6.70[6.25-7.19]
	Dehydration	1318	16907	4.36[4.12-4.61]
	Hyponatremia	2486	24820	5.86[5.62-6.11]
	Anticholinergic syndrome	16	80	12.64[7.31-21.86]
	Hypoglycemia	872	22699	2.04[1.90-21.86]
	Seizure disorder	1028	16204	3.47[3.26-3.70]
	Drug abuse, dependence, withdrawal	423	11880	1.87[1.70-2.06]
	Central nervous system vascular disorders	2228	81993	1.43[1.37-1.49]
	Hearing impairment	465	15789	1.54[1.40-1.69]
	Visual impairment	267	19258	0.71[0.63-0.80]
**Potentially associated prescriptions of drugs known to induce delirium**	Opioids	18384	558493	2.12[2.08-2.16]
	Antipsychotics	5490	83886	3.84[3.73-3.96]
	Anxiolytics	9877	191815	3.16[3.09-3.23]
	Hypnotics	5382	88024	3.56[3.46-3.66]

*** including geographic region**
****434 NA (1% missing data for sex variable)**

Supplementary Table 7 - Sensitivity analysis

		**The event "Delirium" includes "Delirium", "Confusional State" and "Disorientation"**			**The event "Delirium" includes "Delirium" and "Confusional State"**			**The event "Delirium" includes "Delirium", "Confusional State", "Disorientation", and "Circadian rhythm sleep disorder"**			**The event "Delirium" includes "Delirium", "Confusional State", "Disorientation", "Circadian rhythm sleep disorder", and "Hallucination"**		
		Number of delirium reports	Number of all reports	Univariate analysis r-OR[95%CI]	Number of delirium reports	Number of all reports	Univariate analysis r-OR[95%CI]	Number of delirium reports	Number of all reports	Univariate analysis r-OR[95%CI]	Number of delirium reports	Number of all reports	Univariate analysis r-OR[95%CI]
**Total number of reports for 65+ age group**		87524	6048742		78480	6048742		87601	6048742		109504	6048742	
**Antidepressant classes**	Non-selective monoamine reuptake inhibitors	2658	55646	3.49[3.36-3.63]	2474	55646	3.62[3.48-3.77]	2662	55646	3.49[3.36-3.64]	3390	55646	3.60[3.47-3.73]
	Monoamine oxidase inhibitors	158	2256	5.14[4.37-6.04]	150	2256	5.43[4.60-6.41]	158	2256	5.13[4.37-6.03]	186	2256	4.88[4.20-5.67]
	Selective serotonin reuptake inhibitors	6475	148194	3.28[3.20-3.37]	5947	148194	3.36[3.27-3.45]	6481	148194	3.28[3.20-3.37]	8179	148194	3.34[3.27-3.42]
	Serotonin-norepinephrine reuptake inhibitors	2247	54171	3.00[2.87-3.13]	1987	54171	2.95[2.82-3.08]	2250	54171	3.00[2.87-3.13]	2813	54171	3.02[2.91-3.14]
	Alpha-2-adrenergic receptor antagonists	1553	31470	3.58[3.40-3.77]	1436	31470	3.69[3.49-3.89]	1554	31470	3.58[3.40-3.77]	1944	31470	3.62[3.45-3.79]
	Other antidepressants	5850	133197	3.28[3.19-3.37]	5286	133197	3.30[3.21-3.39]	5855	133197	3.28[3.19-3.37]	7542	133197	3.42[3.34-3.51]
